# Crystal structures of six 4-(4-nitro­phenyl)­piperazin-1-ium salts

**DOI:** 10.1107/S2056989022004157

**Published:** 2022-04-26

**Authors:** Ninganayaka Mahesha, Haruvegowda Kiran Kumar, Hemmige S. Yathirajan, Sabine Foro, Mohammed S. M. Abdelbaky, Santiago Garcia-Granda

**Affiliations:** a Department of Studies in Chemistry, University of Mysore, Manasagangotri, Mysore-570 006, India; b Institute of Materials Science, Darmstadt University of Technology, Alarich-Weiss-Strasse 2, D-64287 Darmstadt, Germany; c Department of Physical and Analytical Chemistry, Faculty of Chemistry, Oviedo University-CINN, Oviedo 33006, Spain

**Keywords:** crystal structure, piperazine, benzoate anion, biological activity

## Abstract

Six piperazinium salts, namely 4-(4-nitro­phenyl)­piperazin-1-ium 4-bromo­ben­zo­ate dihydrate, C_10_H_14_N_3_O_2_^+^·C_7_H_4_BrO_2_^−^·2H_2_O, (I), 4-(4-nitro­phenyl)­pi­per­a­zin-1-ium 4-iodo­benzoate dihydrate, C_10_H_14_N_3_O_2_^+^·C_7_H_4_IO_2_^−^·2H_2_O, (II), 4-(4-nitro­phenyl)­piperazin-1-ium 4-hy­droxy­benzoate monohydrate, C_10_H_14_N_3_O_2_^+^·C_7_H_5_O_3_^−^·H_2_O, (III), 4-(4-nitro­phenyl)­piperazin-1-ium 4-methyl­benzoate monohydrate, C_10_H_14_N_3_O_2_^+^·C_8_H_7_O_2_^−^·H_2_O, (IV), 4-(4-nitro­phenyl)­piperazin-1-ium 4-meth­oxy­benzoate hemihydrate, 2C_10_H_14_N_3_O_2_^+^·2C_8_H_7_O_3_^−^·H_2_O, (V), and 4-(4-nitro­phenyl)­piperazin-1-ium 4-eth­oxy­benzoate, 2C_10_H_14_N_3_O_2_^+^·2C_9_H_9_O_3_^−^, (VI), have been synthesized and their crystal structures solved by single-crystal X-ray diffraction, revealing that all of them crystallize in the triclinic space group *P*  except for (V), which crystallizes in the monoclinic space group *P*2_1_/*c* and has a disordered nitro group. Compounds (I) and (II) are isostructural. The crystal packing of (I)–(V) is constructed from organic chains formed by a combination of hydrogen bonds of type N—H⋯O and/or O—H⋯O and other weak inter­actions of type C—H⋯O and/or C—H⋯π, forming sheets, whereas (VI) shows a cationic and anionic-based layer structure.

## Abstract 2

Six piperazinium salts are reported, five of them are hydrated and two crystallized as 2:2 salts. They exhibit asymmetric units of a common 4-nitro­phenyl­piperazine cation and different *p*-substituent benzoate anions. Their crystal structures mainly pack as chains stabilized by strong N—H⋯O and O—H⋯O hydrogen bonds and other weak inter­actions such as C—H⋯O and C—H⋯π.

## 1. Chemical context

Piperazines and substituted piperazines are important pharmacophores that can be found in many biologically active compounds used to treat a number of different diseases (Berkheij, 2005 ▸) as anti­fungal (Upadhayaya *et al.*, 2004 ▸), anti-bacterial, anti-malarial and anti-psychotic agents (Choudhary *et al.*, 2006 ▸). A valuable insight into advances on the anti­microbial activity of piperazine derivatives was given by Kharb *et al.* (2012 ▸). Piperazines are among the most important building blocks in current drug discovery and are found in biologically active compounds across a number of different therapeutic areas (Brockunier *et al.*, 2004 ▸; Bogatcheva *et al.*, 2006 ▸). Pharmacological and toxicological information for piperazine derivatives is reviewed by Elliott (2011 ▸).

4-Nitro­phenyl­piperazinium chloride monohydrate has been used as an inter­mediate in the synthesis of anti­cancer drugs, transcriptase inhibitors and anti­fungal reagents and is also an important reagent for potassium channel openers, which show considerable biomolecular current-voltage rectification characteristics (Lu, 2007 ▸). The inclusion behaviours of 4-sulf­on­ato­calix[n]arenes (SCX*n*) (*n* = 4, 6, 8) with 1-(4-nitrophen­yl)piperazine (NPP) were investigated by UV spectroscopy and fluorescence spectroscopy at different pH values (Zhang *et al.*, 2014 ▸). The design, synthesis and biological profiling of aryl­piperazine-based scaffolds for the management of androgen-sensitive prostatic disorders was reported by Gupta *et al.* (2016 ▸). 4-Nitro­phenyl­piperazine was the starting material in the synthesis and biological evaluation of novel piperazine-containing hydrazone derivatives (Kaya *et al.*, 2016 ▸). The crystal structure of 4-nitro­phenyl piperazinium chloride monohydrate was reported by Lu (2007 ▸) and that of 4,6-di­meth­oxy­pyrimidin-2-amine-1-(4-nitro­phen­yl)piperazine (1:1) by Wang *et al.* (2014 ▸) while Ayeni *et al.* (2019 ▸) described the synthesis and crystal structure of a Schiff base, 5-methyl-2-{[4-(4-nitro­phen­yl)piperazin-1-yl]meth­yl}phenol is published. NMR-based investigations of acyl-functionalized piperazines concerning their conformational behaviour in solution has been studied and the crystal structures of 1-(4-fluoro­benzo­yl)-4-(4-nitro­phen­yl)piperazine, 1-(4-bromo­benzo­yl)-4-(4-nitro­phen­yl)piperazine and 1-(3-bromo­benzo­yl)-4-(4-nitro­phen­yl)piperazine have been reported (Wodtke *et al.*, 2018 ▸). We have recently reported the crystal structures of some salts of 4-meth­oxy­phenyl­piperazine (Kiran Kumar *et al.*, 2019 ▸) and also 2-meth­oxy­phenyl­piperazine (Harish Chinthal *et al.*, 2020 ▸), as well as some salts of piperazine derivatives (Archana *et al.*, 2021 ▸). 
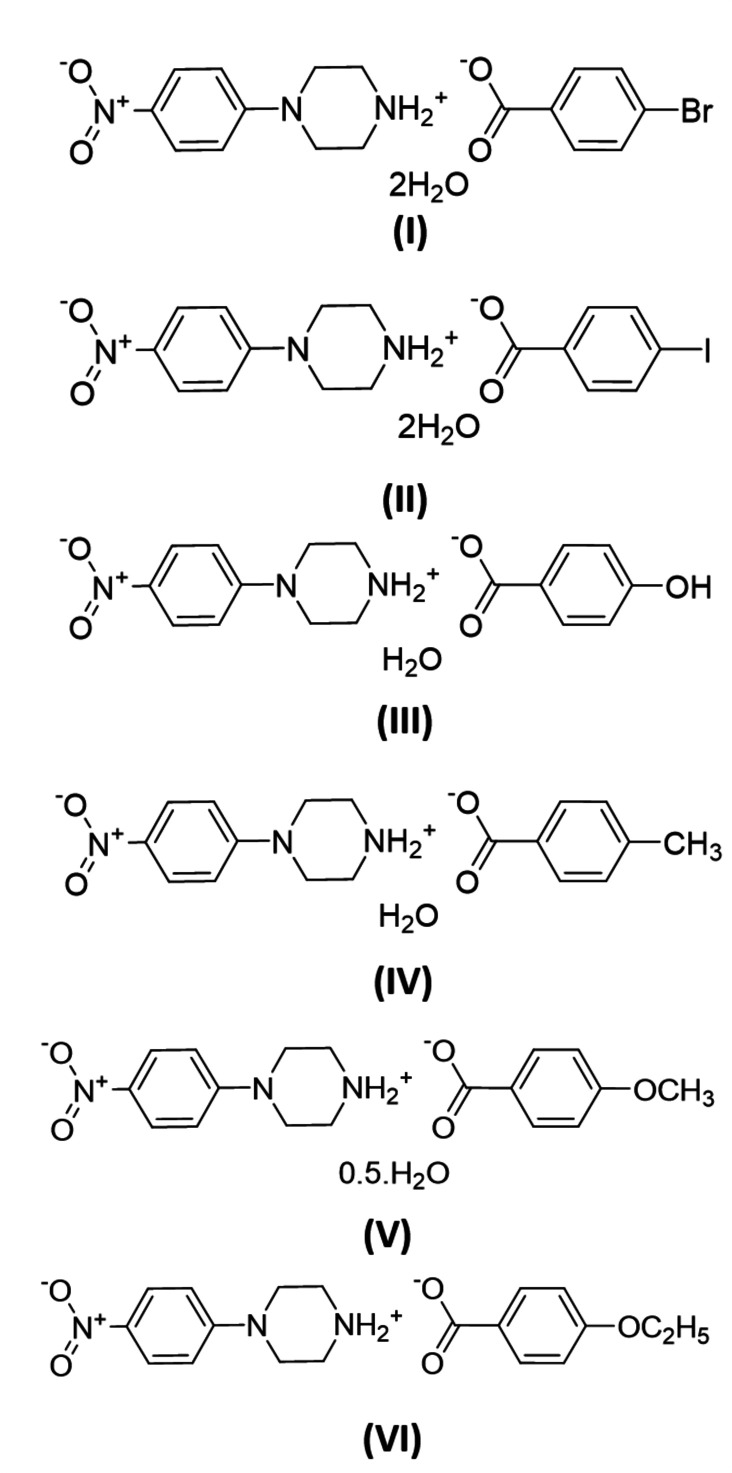


In view of the importance of piperazines in general and the use of 4-nitro­phenyl­piperazine in particular, the present paper reports the crystal structures of some salts of 4-nitro­phenyl­piperazine with organic acids. The crystal structures of 4-(4-nitro­phenyl)­piperazin-1-ium 4-bromo­benzoate dihydrate (I), 4-(4-nitro­phenyl)­piperazin-1-ium 4-iodo­benzoate dihydrate (II), 4-(4-nitro­phenyl)­piperazin-1-ium 4-hy­droxy­ben­zoate monohydrate (III), 4-(4-nitro­phenyl)­piperazin-1-ium 4-methyl­benzoate monohydrate (IV), 4-(4-nitro­phenyl)­pi­per­azin-1-ium 4-meth­oxy­benzoate hemihydrate (V) and 4-(4-nitro­phenyl)­piperazin-1-ium 4-eth­oxy­benzoate (VI) are reported herein.

## 2. Structural commentary

**Figure 1:**
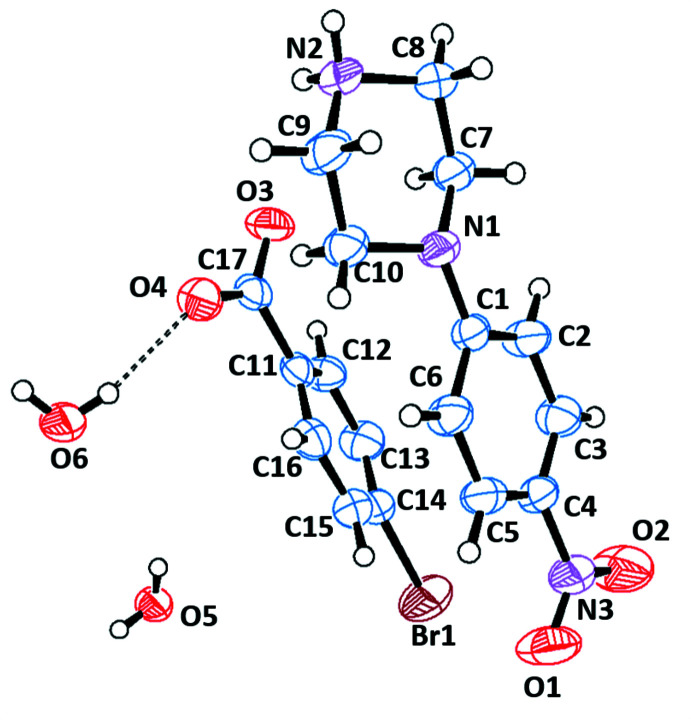
The independent components of compound (I) showing the atom-labelling scheme and the hydrogen bonds, drawn as dashed lines. Displacement ellipsoids are drawn at the 50% probability level.

The asymmetric units of the title salts are shown in Figs. 1 ▸–6 ▸ ▸ ▸ ▸ ▸. They include 1:1 dihydrated salts [(I), (II)], 1:1 monohydrated salts [(III), (IV)], 2:2 monohydrated salt (V) and solvent-free 2:2 salt (VI). Compounds (I) and (II) are isostructural. In all salts, the cation is common and consists of a protonated chair-shaped piperazine ring (N1/N2/C7–C10), which makes dihedral angles of 10.91 (1), 12.13 (1), 14.82 (6), 3.11 (8), 5.73 (1) and 13.08 (9)°, respectively, for compounds (I)–(VI) with the nitro­benzene moiety (N3/O1/O2/C1–C6) and exhibits a maximum deviation from its mean plane at atom N2 of −0.253 (2), 0.254 (2), 0.288 (2), 0.278 (2), 0.241 (3) and 0.303 (3) Å in (I)–(VI), respectively. The piperazine rings of the additional cations (N4/N5/C25–C28) in compounds (V) and (VI) have the same conformation, making dihedral angles of 64.53 (1) and 21.70 (1)°, respectively, with the nitro­benzene moieties (N6/O6/O7/C19–C25). Within the cations, the benzene rings are almost planar, with maximum deviations from mean plane ranging from −0.016 (3) Å at atom C20 for (VI) to 0.003 (2) Å at atom C4 for (III). The *p*-nitro substituent groups deviate significantly from planes of the benzene rings in all compounds except the (C1–C6) ring of (VI). The anions of the title salts are formed from a benzoate anion with different *p*-substituents for each compound that deviate significantly from planarity, with maximum deviations of 0.045 (1) Å at Br1 for (I), 0.063 (1) Å at I1 for (II), −0.021 (2) Å at hydroxyl atom O3 for (III), −0.010 (1) Å at methyl atom C18 for (IV), −0.033 (1) and 0.034 (1) Å at meth­oxy atoms O5 and O10 for (V) and −0.025 (2) and −0.013 (2) Å at eth­oxy atoms O5 and O10 for (VI).

## 3. Supra­molecular features

**Figure 7:**
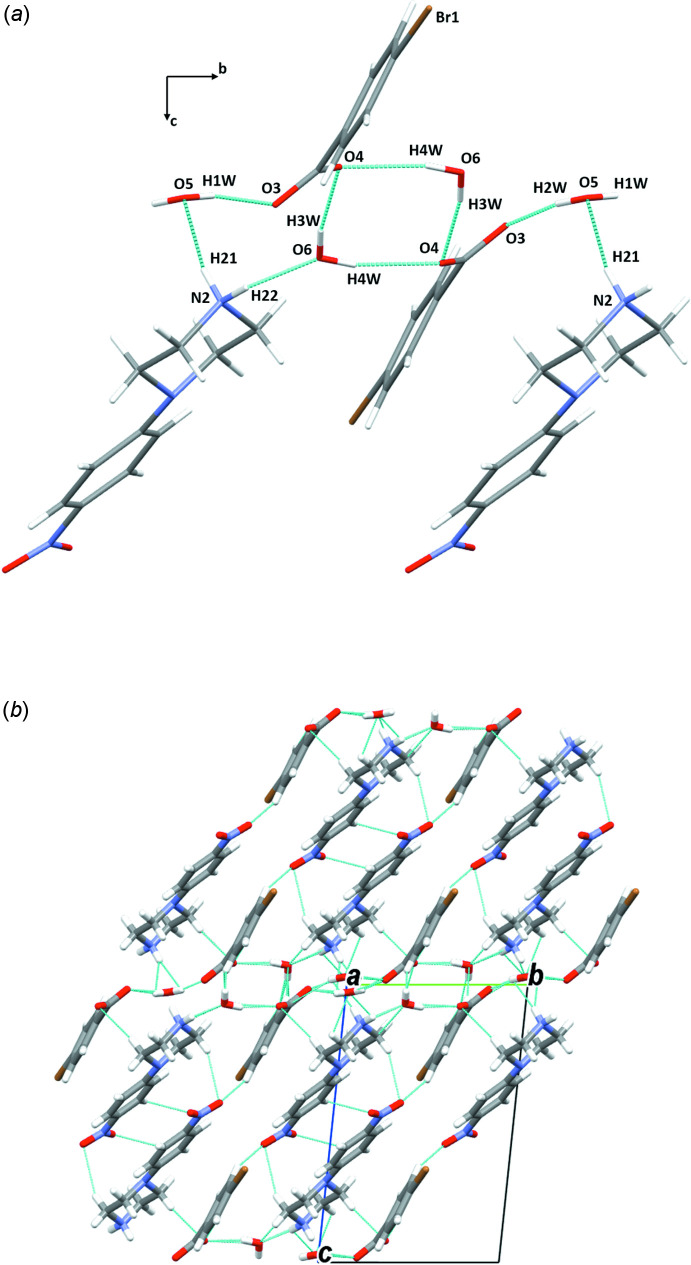
(*a*) A general view of the main inter­molecular inter­actions (N—H⋯O and O—H⋯O) and (*b*) the mol­ecular packing of (I) with hydrogen bonds shown as dashed lines.

**Figure 9:**
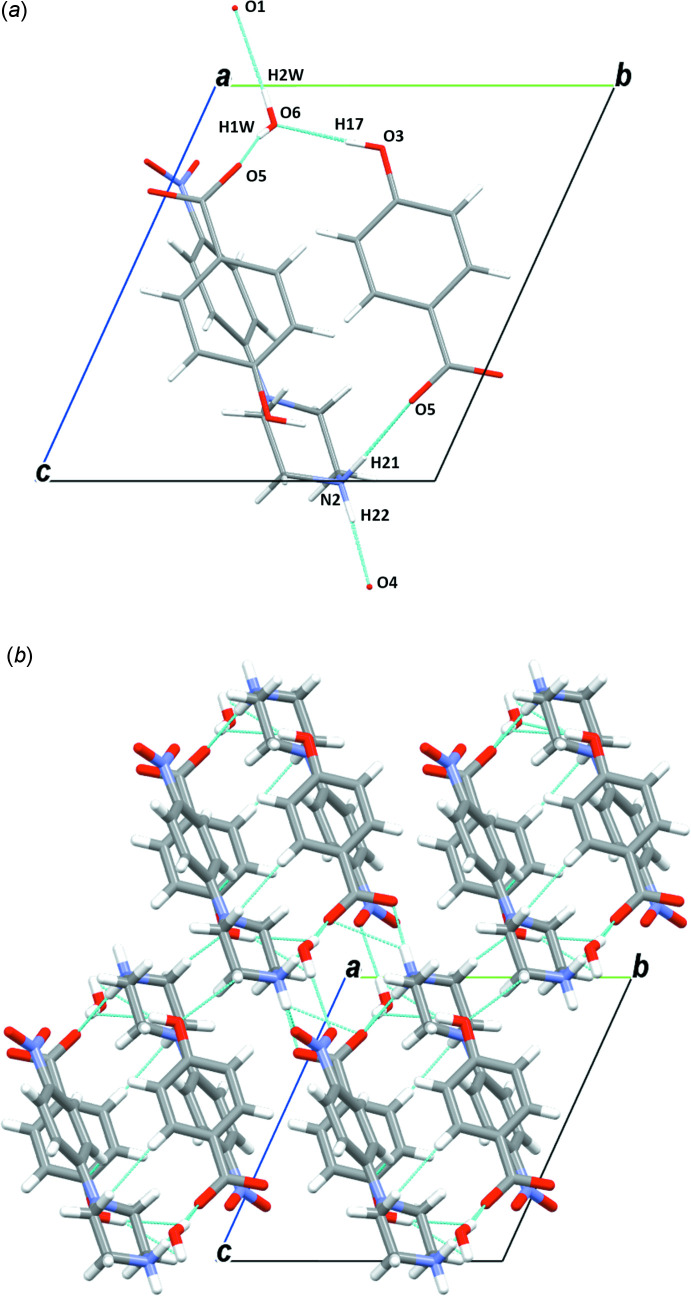
(*a*) A general view of the main inter­molecular inter­actions (N—H⋯O and O—H⋯O) in (III) and (*b*) the mol­ecular packing of (III) with hydrogen bonds shown as dashed lines.

**Figure 10:**
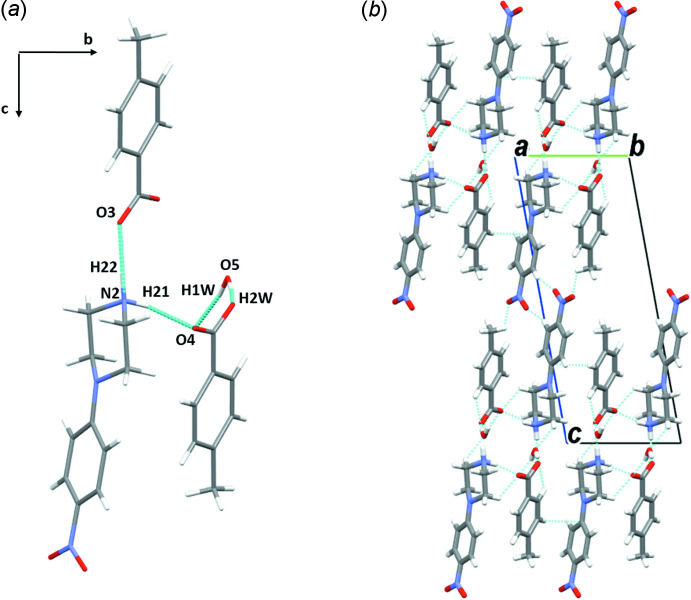
(*a*) A general view of the main inter­molecular inter­actions (N—H⋯O and O—H⋯O) in (IV) and (*b*) the mol­ecular packing of (IV) with hydrogen bonds shown as dashed lines.

**Figure 11:**
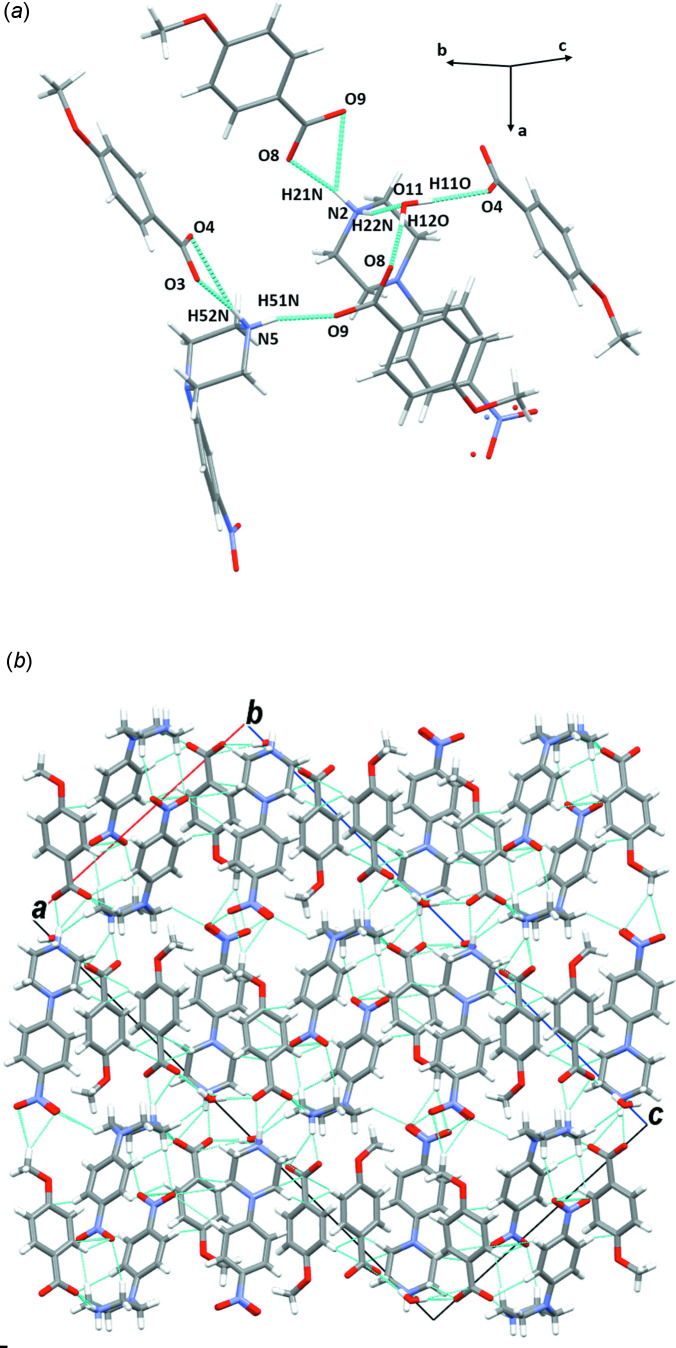
(*a*) A general view of the main inter­molecular inter­actions (N—H⋯O and O—H⋯O) in (V) and (*b*) the mol­ecular packing of (V) with hydrogen bonds shown as dashed lines.

**Figure 12:**
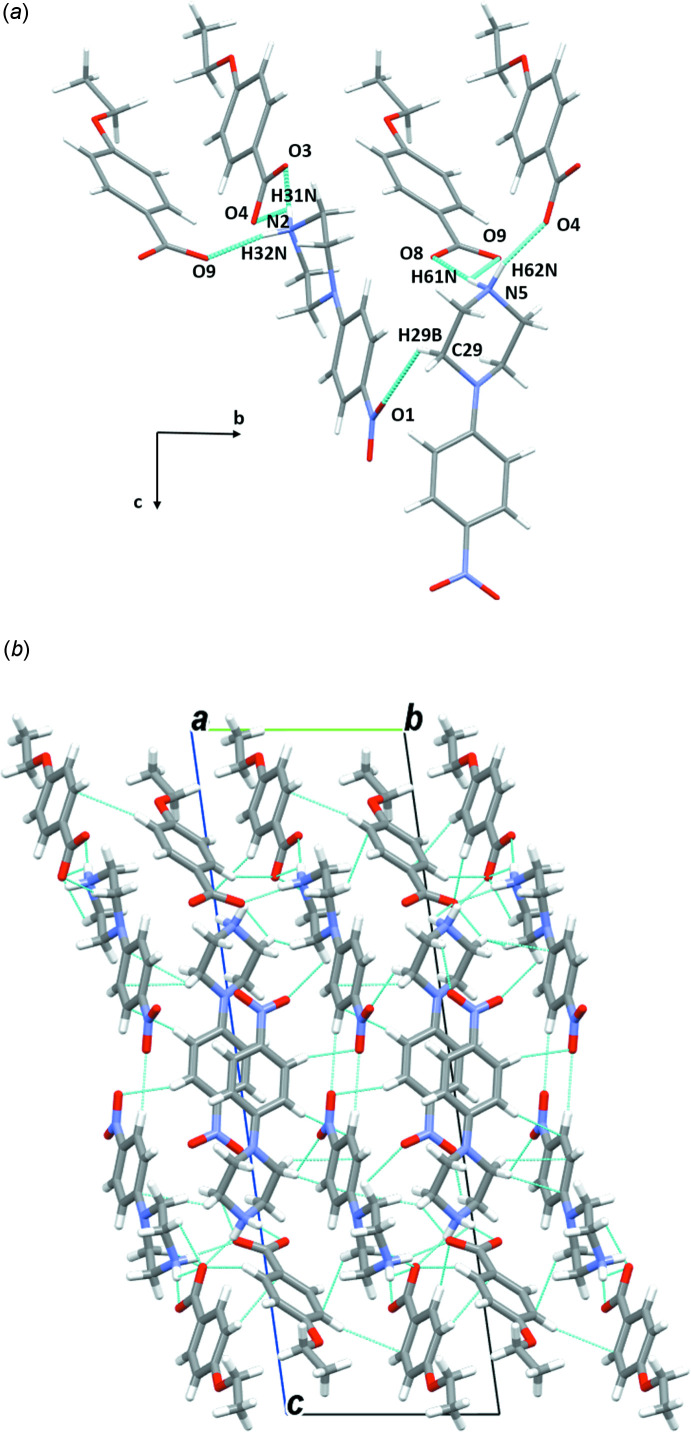
(*a*) A general view of the main inter­molecular inter­actions (N—H⋯O, O—H⋯O and C—H⋯O) in (VI) and (*b*) the mol­ecular packing of (VI) with hydrogen bonds shown as dashed lines.

In the crystal structures of the two isomorphous salts (I) and (II), the ions are arranged in chains perpendicular to the *a-*axis direction. The water mol­ecules play an essential role in holding the chains together, forming complex sheets in the *bc* plane (Figs. 7 ▸ and 8 ▸, Tables 1 ▸ and 2 ▸). The cations and anions in (III) are linked through strong O—H⋯O and N—H⋯O hydrogen bonds, forming chains along the [011] direction (Fig. 9 ▸ *a*, Table 3 ▸). These chains are further linked *via* the water mol­ecules and C9—H9*A*⋯O3 inter­actions, generating a three-dimensional supra­molecular architecture along the *a* axis (Fig. 9 ▸ *b*). The structure of (IV) is constructed from double chains running along the [101] direction. Each chain is formed by linking the mol­ecules through a combination of N—H⋯O, O—H⋯O and C—H⋯O inter­actions (Fig. 10 ▸ *a*, Table 4 ▸); the resulting double chains are symmetrically related by an inversion center and are connected *via* N2—H21⋯O4 and C7—H7*A*⋯O4 inter­actions. These hydrated double chains are weakly linked into sheets lying in the *bc* plane by C—H⋯π (arene) inter­actions (Fig. 10 ▸ *b*). The supra­molecular assembly of compound (V), which has a disordered nitro group, is built up of N2—H22*N*⋯O11, O11—H11*O*⋯O4 and N5—H51⋯O9 hydrogen bonds linking the ions into organic chains running parallel to the [010] direction (Fig. 11 ▸ *a*, Table 5 ▸). The chains are further connected cooperatively through other inter­actions of type N—H⋯O, generating a multilayer network along the *b-*axis direction (Fig. 11 ▸ *b*). In compound (VI), a set of N—H⋯O, C—H⋯O and C—H⋯π inter­actions (Fig. 12 ▸ *a*, Table 6 ▸) link the mol­ecules into cationic and anionic layers running parallel to the *b-*axis direction and join these layer motifs, generating the complete mol­ecular structure along the *a* axis (Fig. 12 ▸ *b*).

## 4. Database survey

A search of the Cambridge Structural Database (Version 2020.3, last update February 2022; Groom *et al.*, 2016 ▸) for the phenyl piperazinium cation and *para* substituent benzoate anion involved in the reported six salts gave the following hits, 4-(4-meth­oxy­phen­yl)piperazin-1-ium 4-fluoro­benzoate mono­hydrate, 4-(4-meth­oxy­phen­yl)piperazin-1-ium 4-chloro­benzoate monohydrate and 4-(4-meth­oxy­phen­yl)piperazin-1-ium 4-bromo­benzoate monohydrate (FOVPOY, FOVPUE and FOVQAL; Kiran Kumar *et al.*, 2019 ▸) and 4-(4-meth­oxy­phen­yl)piperazin-1-ium 4-iodo­benzoate monohydrate (KUJ­PUD; Kiran Kumar *et al.*, 2020 ▸). They exhibit a meth­oxy group as a substituent in the phenyl piperazinium cation rather than a nitro group as in the title compounds (I)–(VI) and they also crystallize as monohydrates similar to compounds (III)–(V). Although the title compounds (I) and (II) have halogen-based anions and chain-based structures, they are not isostructural with the above compounds, the crystal structures of which are based on differently sized chains of rings formed *via* a combination of hydrogen bonds of type N—H⋯O and O—H⋯O and other weak inter­actions of type C—H⋯O and C—H⋯π to form sheets. In 4-(4-meth­oxy­phen­yl)piperazin-1-ium 4-amino­benzoate monohydrate (IHIMEU; Kiran Kumar *et al.*, 2020 ▸) the presence of an amino substituent on the anion, which acts as both a donor and an acceptor of hydrogen bonds, makes the supra­molecular assembly of this compound more complex than for the compounds reported herein.

## 5. Synthesis and crystallization

**Synthesis:**

For the synthesis of salts (I)–(VI), a solution of commercially available (from Sigma–Aldrich) 4-nitro­phenyl­piperazine (100 mg, 0.483 mol) in methanol (10 ml) was mixed with equimolar solutions of the appropriate acids in methanol (10 ml) and ethyl acetate (10 ml), *viz*.4-bromo­benzoic acid (97 mg, 0.483 mol) for (I), 4-iodo­benzoic acid (120 mg, 0.483 mol) for (II), 4-hy­droxy­benzoic acid (67 mg, 0.483 mol) for (III), 4-methyl­benzoic acid (66 mg, 0.483 mol) for (IV), 4-meth­oxy­benzoic acid (73 mg, 0.483 mol) for (V) and 4-eth­oxy­benzoic­acid (80 mg, 0.483 mol) for (VI). The corres­ponding solutions were stirred for 15 minutes at room temperature and allowed to stand at the same temperature. The products obtained were subjected to crystallization.

**Crystallization:** Crystallization was carried out using the slow evaporation technique. X-ray quality crystals were formed on slow evaporation in a week for all compounds, where ethanol:ethyl­acetate (1:1) was used for crystallization. The corresponding melting points were 430–432 K (I), 453–455 K (II), 446–448 K (III), 398–400 K (IV), 413–415 K (V) and 408–410 K (VI).

## 6. Refinement

Crystal data, data collection and refinement details are summarized in Table 7 ▸. C-bound H atoms were positioned with idealized geometry and refined using a riding model with C—H = 0.93 Å (aromatic), 0.96 Å (meth­yl) or 0.97 Å (methyl­ene). The H atoms on the N atom were located in a difference map and later restrained to N—H = 0.86 (2) Å. All H atoms were refined with isotropic displacement parameters set at 1.2 *U*_eq_ (C-aromatic, C-methyl­ene, N) or 1.5 *U*_eq_ (C-meth­yl) times those of the parent atom. For the disordered nitro group in (V), the component atoms were restrained to have the same *U^ij^* components and the occupancy ratio is 0.519 (6):0.481 (6).

## Supplementary Material

Crystal structure: contains datablock(s) global, I, II, III, IV, V, VI. DOI: 10.1107/S2056989022004157/ex2056sup1.cif

Structure factors: contains datablock(s) I. DOI: 10.1107/S2056989022004157/ex2056Isup2.hkl

Structure factors: contains datablock(s) II. DOI: 10.1107/S2056989022004157/ex2056IIsup3.hkl

Structure factors: contains datablock(s) III. DOI: 10.1107/S2056989022004157/ex2056IIIsup4.hkl

Structure factors: contains datablock(s) IV. DOI: 10.1107/S2056989022004157/ex2056IVsup5.hkl

Structure factors: contains datablock(s) V. DOI: 10.1107/S2056989022004157/ex2056Vsup6.hkl

Structure factors: contains datablock(s) VI. DOI: 10.1107/S2056989022004157/ex2056VIsup7.hkl

CCDC references: 2167424, 2167423, 2167422, 2167421, 2167420, 2167419

Additional supporting information:  crystallographic information; 3D view; checkCIF report

## Figures and Tables

**Figure 2:**
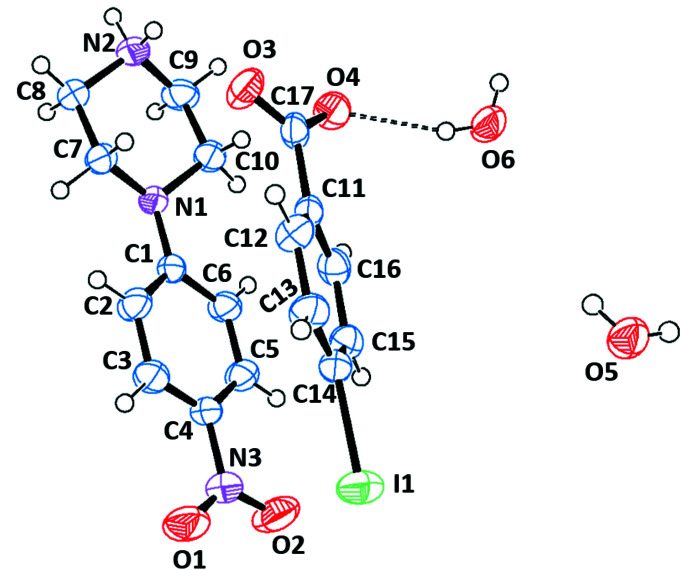
The independent components of compound (II) showing the atom-labelling scheme and the hydrogen bonds, drawn as dashed lines. Displacement ellipsoids are drawn at the 50% probability level.

**Figure 3:**
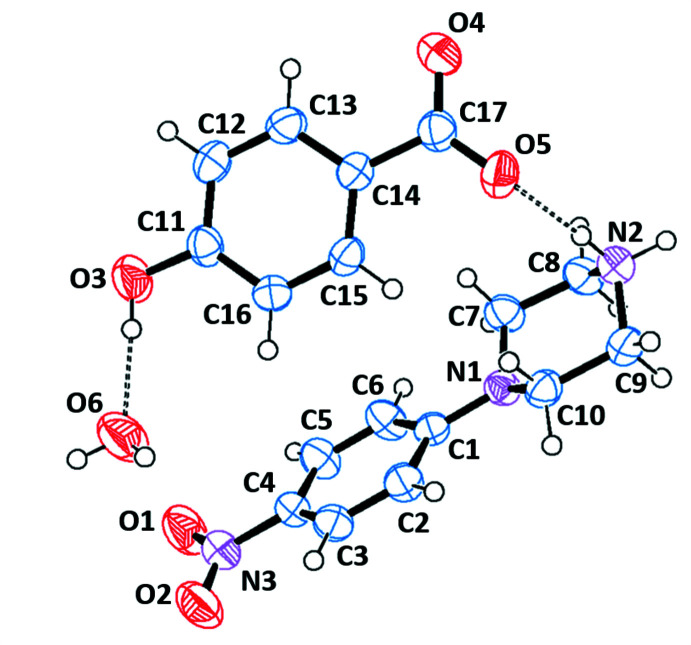
The independent components of compound (III) showing the atom-labelling scheme and the hydrogen bonds, drawn as dashed lines. Displacement ellipsoids are drawn at the 50% probability level.

**Figure 4:**
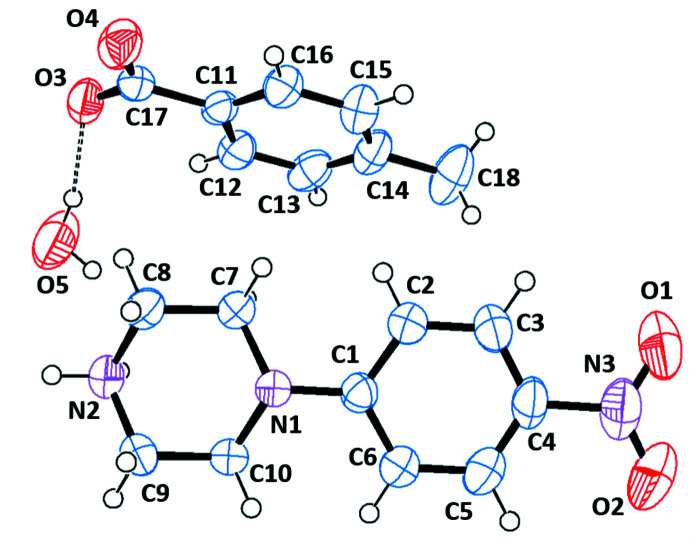
The independent components of compound (IV) showing the atom-labelling scheme and the hydrogen bonds, drawn as dashed lines. Displacement ellipsoids are drawn at the 50% probability level.

**Figure 5:**
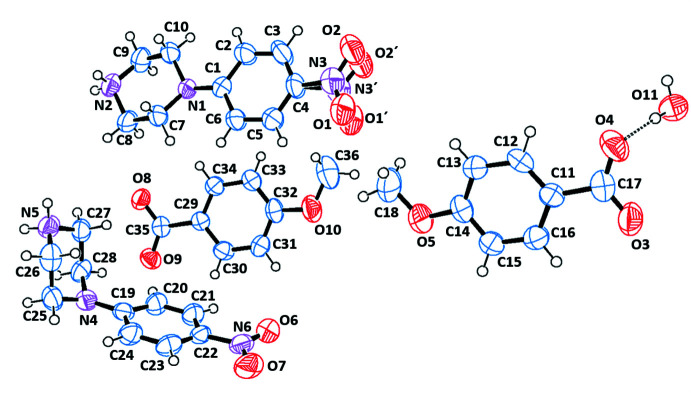
The independent components of compound (V) showing the atom-labelling scheme and the hydrogen bonds, drawn as dashed lines. Displacement ellipsoids are drawn at the 50% probability level.

**Figure 6:**
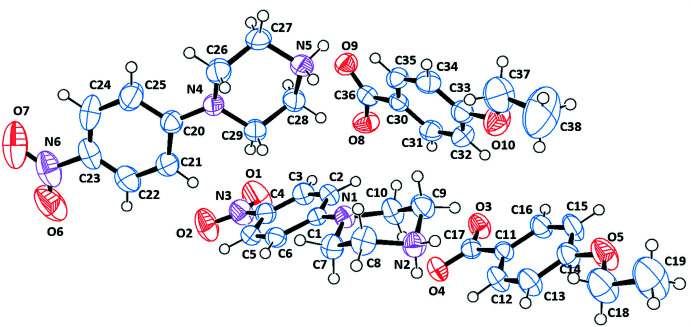
The independent components of compound (VI) showing the atom-labelling scheme Displacement ellipsoids are drawn at the 50% probability level.

**Figure 8:**
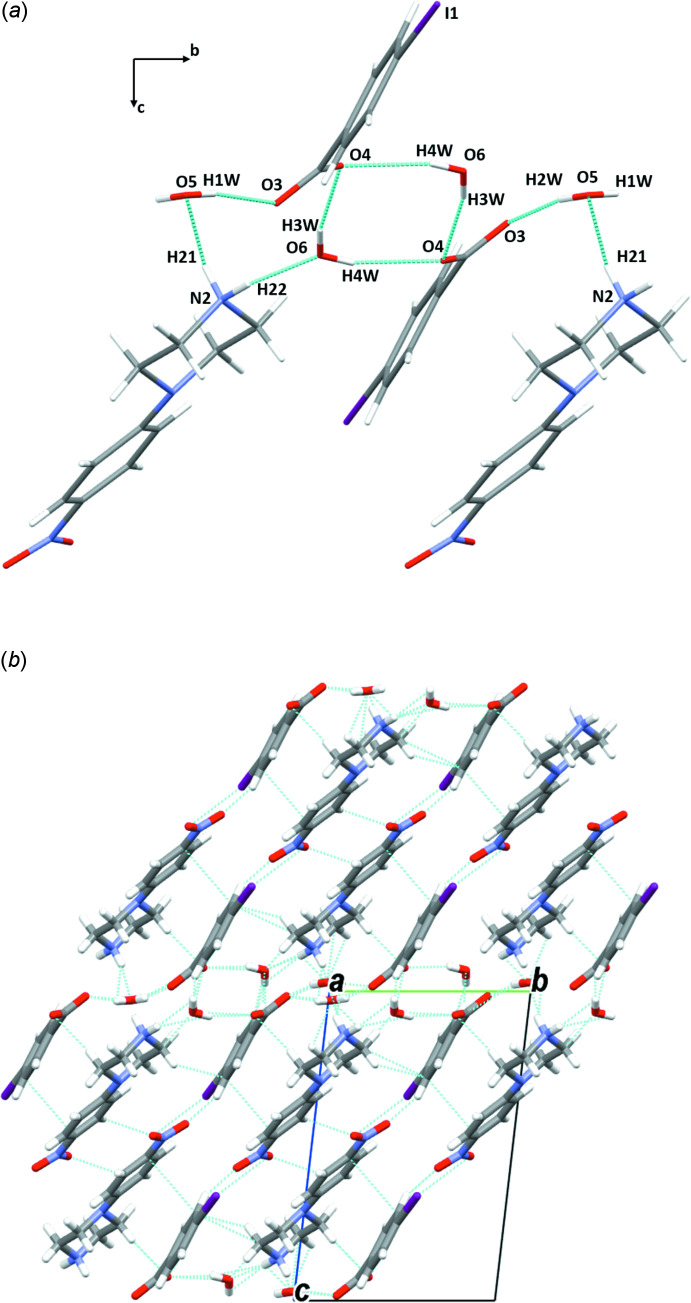
(*a*) A general view of the main inter­molecular inter­actions (N—H⋯O and O—H⋯O) in (II) and (*b*) the mol­ecular packing of (II) with hydrogen bonds shown as dashed lines.

**Table 1:** Hydrogen-bond geometry (Å, °) for (I)

*D*—H⋯*A*	*D*—H	H⋯*A*	*D*⋯*A*	*D*—H⋯*A*
N2—H21⋯O5^i^	0.85 (2)	1.99 (2)	2.810 (3)	162 (3)
N2—H22⋯O6^ii^	0.83 (2)	1.91 (2)	2.707 (3)	160 (3)
C3—H3⋯O1^iii^	0.93	2.59	3.260 (4)	130
C13—H13⋯O4^iv^	0.93	2.57	3.483 (3)	166
C15—H15⋯O2^v^	0.93	2.47	3.269 (4)	144
O5—H1*W*⋯O3^vi^	0.80 (2)	1.97 (2)	2.759 (2)	169 (3)
O5—H2*W*⋯O3^i^	0.80 (2)	2.00 (2)	2.772 (2)	161 (3)
O6—H4*W*⋯O4	0.82 (2)	2.03 (2)	2.832 (3)	166 (3)
O6—H3*W*⋯O4^vii^	0.78 (2)	1.99 (2)	2.760 (3)	169 (3)

Symmetry codes: (i) 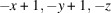 ; (ii) 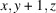 ; (iii) 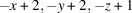 ; (iv) 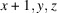 ; (v) 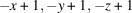 ; (vi) 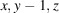 ; (vii) 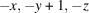 .

**Table 2:** Hydrogen-bond geometry (Å, °) for (II)

*D*—H⋯*A*	*D*—H	H⋯*A*	*D*⋯*A*	*D*—H⋯*A*
N2—H21⋯O5^i^	0.86 (2)	1.99 (2)	2.825 (4)	164 (4)
N2—H22⋯O6^ii^	0.85 (2)	1.88 (2)	2.702 (3)	163 (4)
C3—H3⋯O1^iii^	0.93	2.59	3.275 (4)	131
C13—H13⋯O4^iv^	0.93	2.62	3.526 (4)	166
C15—H15⋯O2^v^	0.93	2.49	3.311 (4)	147
O5—H1*W*⋯O3^vi^	0.81 (2)	1.96 (2)	2.756 (3)	170 (4)
O5—H2*W*⋯O3^i^	0.81 (2)	1.96 (2)	2.753 (3)	166 (4)
O6—H4*W*⋯O4	0.80 (2)	2.08 (2)	2.836 (3)	160 (4)
O6—H3*W*⋯O4^vii^	0.80 (2)	1.95 (2)	2.728 (3)	165 (4)

Symmetry codes: (i) 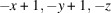 ; (ii) 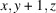 ; (iii) 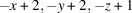 ; (iv) 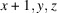 ; (v) 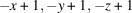 ; (vi) 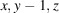 ; (vii) 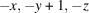 .

**Table 3:** Hydrogen-bond geometry (Å, °) for (III)

*D*—H⋯*A*	*D*—H	H⋯*A*	*D*⋯*A*	*D*—H⋯*A*
N2—H21⋯O5	0.89 (2)	1.93 (2)	2.819 (2)	177 (3)
N2—H22⋯O4^i^	0.94 (2)	1.65 (2)	2.583 (2)	177 (3)
O3—H17⋯O6	0.85 (2)	1.82 (2)	2.669 (2)	177 (3)
O6—H1*W*⋯O5^ii^	0.83 (2)	1.95 (2)	2.768 (2)	169 (3)
O6—H2*W*⋯O1^iii^	0.83 (2)	2.11 (2)	2.944 (2)	178 (3)

Symmetry codes: (i) 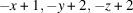 ; (ii) 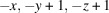 ; (iii) 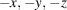 .

**Table 4:** Hydrogen-bond geometry (Å, °) for (IV) *Cg*3 is the centroids of the C11–C16 ring.

*D*—H⋯*A*	*D*—H	H⋯*A*	*D*⋯*A*	*D*—H⋯*A*
N2—H21⋯O4^i^	0.89 (2)	1.93 (2)	2.811 (3)	167 (4)
N2—H22⋯O3^ii^	0.91 (2)	1.81 (2)	2.717 (3)	177 (4)
C3—H3⋯O1^iii^	0.93	2.54	3.427 (4)	161
C9—H9*A*⋯O5^iv^	0.97	2.31	3.113 (3)	140
O5—H1*W*⋯O4^i^	0.84 (2)	1.92 (2)	2.756 (3)	171 (4)
O5—H2*W*⋯O3	0.85 (2)	1.94 (2)	2.772 (3)	164 (4)
C6—H6⋯*Cg*3^v^	0.93	2.93	3.590 (3)	129

Symmetry codes: (i) 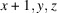 ; (ii) 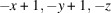 ; (iii) 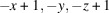 ; (iv) 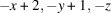 ; (v) 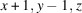 .

**Table 5:** Hydrogen-bond geometry (Å, °) for (V)

*D*—H⋯*A*	*D*—H	H⋯*A*	*D*⋯*A*	*D*—H⋯*A*
C7—H7*B*⋯O7^i^	0.97	2.54	3.451 (5)	157
C9—H9*B*⋯O4^ii^	0.97	2.31	3.270 (5)	169
C20—H20⋯O9	0.93	2.53	3.461 (5)	174
C25—H25*A*⋯O2a^iii^	0.97	2.5	3.206 (10)	130
C25—H25*A*⋯O2′b^iii^	0.97	2.49	3.212 (11)	131
C27—H27*A*⋯O7^i^	0.97	2.58	3.548 (5)	175
C28—H28*B*⋯O9	0.97	2.55	3.489 (5)	164
C36—H36*C*⋯O1′b^ii^	0.96	2.49	3.395 (14)	158
N2—H21*N*⋯O8^iv^	0.88 (2)	1.83 (2)	2.697 (4)	166 (4)
N2—H21*N*⋯O9^iv^	0.88 (2)	2.57 (3)	3.196 (4)	129 (3)
N2—H22*N*⋯O11^v^	0.88 (2)	1.89 (2)	2.758 (5)	169 (4)
N5—H51*N*⋯O9^vi^	0.87 (2)	1.93 (2)	2.778 (5)	164 (4)
N5—H52*N*⋯O3^vii^	0.91 (2)	1.82 (2)	2.724 (5)	171 (4)
O11—H11*O*⋯O4	0.84 (2)	1.83 (2)	2.663 (4)	176 (4)
O11—H12*O*⋯O8^v^	0.84 (2)	1.92 (2)	2.754 (4)	173 (4)

Symmetry codes: (i) 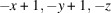 ; (ii) 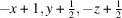 ; (iii) 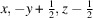 ; (iv) 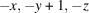 ; (v) 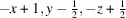 ; (vi) 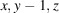 ; (vii) 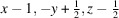 .

**Table 6:** Hydrogen-bond geometry (Å, °) for (VI) *Cg*2 and *Cg*6 are the centroids of the C1–C6 and C30–C35 rings, respectively.

*D*—H⋯*A*	*D*—H	H⋯*A*	*D*⋯*A*	*D*—H⋯*A*
N2—H31*N*⋯O3^i^	0.94 (2)	1.68 (2)	2.613 (3)	172 (5)
N2—H31*N*⋯O4^i^	0.94 (2)	2.51 (4)	3.157 (3)	127 (4)
N2—H32*N*⋯O9^ii^	0.90 (2)	1.96 (2)	2.843 (3)	171 (5)
N5—H61*N*⋯O8^i^	0.91 (2)	1.78 (2)	2.686 (3)	175 (5)
N5—H61*N*⋯O9^i^	0.91 (2)	2.59 (4)	3.174 (3)	122 (4)
N5—H62*N*⋯O4^iii^	0.90 (2)	1.83 (2)	2.708 (3)	165 (5)
C22—H22⋯O2^iv^	0.93	2.6	3.502 (5)	165
C27—H27*B*⋯O9^i^	0.97	2.59	3.215 (3)	123
C28—H28*B*⋯O7^v^	0.97	2.65	3.410 (4)	135
C29—H29*B*⋯O1^i^	0.97	2.53	3.249 (4)	131
C35—H35⋯O4^iii^	0.93	2.52	3.263 (3)	137
C10—H10*A*⋯*Cg*6	0.97	2.82	3.746 (3)	159
C29—H29*A*⋯*Cg*2	0.97	2.76	3.556 (3)	139

Symmetry codes: (i) 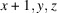 ; (ii) 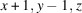 ; (iii) 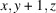 ; (iv) 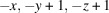 ; (v) 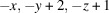 .

**Table:** 

	(I)	(II)	(III)
Crystal data			
Chemical formula	C_10_H_14_N_3_O_2_^+^·C_7_H_4_BrO_2_^−^·2H_2_O	C_10_H_14_N_3_O_2_^+^·C_7_H_4_IO_2_^−^·2H_2_O	C_10_H_14_N_3_O_2_^+^·C_7_H_5_O_3_^−^·H_2_O
*M*_r_	444.28	491.28	363.37
Crystal system, space group	Triclinic, *P* 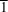	Triclinic, *P* 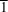	Triclinic, *P* 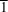
Temperature (K)	293	293	293
*a*, *b*, *c* (Å)	7.738 (1), 9.320 (1), 13.949 (2)	7.7652 (4), 9.2852 (5), 13.930 (1)	9.636 (1), 10.301 (1), 10.867 (1)
α, β, γ (°)	94.46 (1), 95.04 (1), 104.71 (2)	94.985 (5), 95.331 (5), 104.875 (6)	103.90 (1), 108.32 (1), 112.96 (1)
*V* (Å^3^)	964.0 (2)	960.09 (10)	857.80 (17)
*Z*	2	2	2
Radiation type	Mo *K*α	Mo *K*α	Mo *K*α
μ (mm^−1^)	2.17	1.71	0.11
Crystal size (mm)	0.48 × 0.44 × 0.24	0.48 × 0.48 × 0.2	0.50 × 0.32 × 0.24

Data collection			
Diffractometer	Oxford Diffraction Xcalibur	Oxford Diffraction Xcalibur	Oxford Diffraction Xcalibur
Absorption correction	Multi-scan (*CrysAlis RED*; Oxford Diffraction, 2009 ▸)	Multi-scan (*CrysAlis RED*; Oxford Diffraction, 2009 ▸)	Multi-scan (*CrysAlis RED*; Oxford Diffraction, 2009 ▸)
*T*_min_, *T*_max_	0.367, 0.422	0.458, 0.711	0.959, 0.974
No. of measured, independent and observed [*I* > 2σ(*I*)] reflections	6123, 3536, 2520	6331, 3518, 2952	5342, 3140, 2342
*R*_int_	0.019	0.017	0.013
(sin θ/λ)_max_ (Å^−1^)	0.602	0.602	0.602

Refinement			
*R*[*F* ^2^ > 2σ(*F* ^2^)], *wR*(*F* ^2^), *S*	0.037, 0.104, 1.04	0.029, 0.069, 1.03	0.043, 0.106, 1.05
No. of reflections	3528	3513	3135
No. of parameters	262	262	251
No. of restraints	6	6	5
H-atom treatment	H atoms treated by a mixture of independent and constrained refinement	H atoms treated by a mixture of independent and constrained refinement	H atoms treated by a mixture of independent and constrained refinement
Δρ_max_, Δρ_min_ (e Å^−3^)	0.50, −0.51	0.54, −0.66	0.19, −0.19

**Table:** 

	(IV)	(V)	(VI)
Crystal data			
Chemical formula	C_10_H_14_N_3_O_2_^+^·C_8_H_7_O_2_^−^·H_2_O	2C_10_H_14_N_3_O_2_^+^·2C_8_H_7_O_3_^−^·H_2_O	C_10_H_14_N_3_O_2_^+^·C_9_H_9_O_3_^−^
*M*_r_	361.39	736.77	373.4
Crystal system, space group	Triclinic, *P* 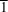	Monoclinic, *P*2_1_/*c*	Triclinic, *P* 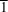
Temperature (K)	293	293	293
*a*, *b*, *c* (Å)	6.1136 (5), 7.6965 (7), 19.708 (2)	15.808 (1), 7.5198 (7), 31.020 (2)	7.874 (1), 9.263 (1), 27.996 (3)
α, β, γ (°)	79.577 (8), 87.162 (8), 86.699 (8)	90, 92.561 (7), 90	81.030 (6), 85.675 (6), 68.229 (5)
*V* (Å^3^)	909.79 (15)	3683.8 (5)	1872.8 (4)
*Z*	2	4	4
Radiation type	Mo *K*α	Mo *K*α	Mo *K*α
μ (mm^−1^)	0.10	0.1	0.10
Crystal size (mm)	0.48 × 0.26 × 0.02	0.5 × 0.36 × 0.36	0.44 × 0.32 × 0.08

Data collection			
Diffractometer	Oxford Diffraction Xcalibur	Oxford Diffraction Xcalibur	Oxford Diffraction Xcalibur
Absorption correction	Multi-scan (*CrysAlis RED*; Oxford Diffraction, 2009 ▸)	Multi-scan (*CrysAlis RED*; Oxford Diffraction, 2009 ▸)	Multi-scan (*CrysAlis RED*; Oxford Diffraction, 2009 ▸)
*T*_min_, *T*_max_	0.970, 0.998	0.958, 0.965	0.963, 0.992
No. of measured, independent and observed [*I* > 2σ(*I*)] reflections	5980, 3347, 1911	15326, 6718, 2602	13344, 6868, 3803
*R*_int_	0.019	0.066	0.027
(sin θ/λ)_max_ (Å^−1^)	0.602	0.602	0.602

Refinement			
*R*[*F* ^2^ > 2σ(*F* ^2^)], *wR*(*F* ^2^), *S*	0.053, 0.138, 1.01	0.074, 0.169, 1.00	0.061, 0.137, 1.05
No. of reflections	3343	6715	6858
No. of parameters	248	507	501
No. of restraints	4	45	16
H-atom treatment	H atoms treated by a mixture of independent and constrained refinement	H atoms treated by a mixture of independent and constrained refinement	H atoms treated by a mixture of independent and constrained refinement
Δρ_max_, Δρ_min_ (e Å^−3^)	0.20, −0.16	0.27, −0.18	0.23, −0.22

Computer programs: *CrysAlis CCD* (Oxford Diffraction, 2009 ▸), *CrysAlis RED* (Oxford Diffraction, 2009 ▸), *SHELXT* (Sheldrick, 2015*a* ▸), *Mercury* (Macrae *et al.*, 2020 ▸), *SHELXL2014* (Sheldrick, 2015*b* ▸), *PLATON* (Spek, 2020 ▸) and *publCIF* (Westrip, 2010 ▸).

## supplementary crystallographic information

### 4-(4-Nitrophenyl)piperazin-1-ium 4-bromobenzoate dihydrate (I) Crystal data

**Table:**

C_10_H_14_N_3_O_2_^+^·C_7_H_4_BrO_2_^−^·2H_2_O	*Z* = 2
*M**_r_* = 444.28	*F*(000) = 456
Triclinic, *P*1	*D*_x_ = 1.531 Mg m^−^^3^
Hall symbol: -P 1	Mo *K*α radiation, λ = 0.71073 Å
*a* = 7.738 (1) Å	Cell parameters from 6123 reflections
*b* = 9.320 (1) Å	θ = 3.0–25.3°
*c* = 13.949 (2) Å	µ = 2.17 mm^−^^1^
α = 94.46 (1)°	*T* = 293 K
β = 95.04 (1)°	Prism, yellow
γ = 104.71 (2)°	0.48 × 0.44 × 0.24 mm
*V* = 964.0 (2) Å^3^	

### 4-(4-Nitrophenyl)piperazin-1-ium 4-bromobenzoate dihydrate (I) Data collection

**Table:**

Oxford Diffraction Xcalibur diffractometer	2520 reflections with *I* > 2σ(*I*)
ω scans	*R*_int_ = 0.019
Absorption correction: multi-scan (CrysAlis RED; Oxford Diffraction, 2009)	θ_max_ = 25.3°, θ_min_ = 3.0°
*T*_min_ = 0.367, *T*_max_ = 0.422	*h* = −9→8
6123 measured reflections	*k* = −6→11
3536 independent reflections	*l* = −16→16

### 4-(4-Nitrophenyl)piperazin-1-ium 4-bromobenzoate dihydrate (I) Refinement

**Table:**

Refinement on *F*^2^	Primary atom site location: structure-invariant direct methods
Least-squares matrix: full	Secondary atom site location: structure-invariant direct methods
*R*[*F*^2^ > 2σ(*F*^2^)] = 0.037	Hydrogen site location: mixed
*wR*(*F*^2^) = 0.104	H atoms treated by a mixture of independent and constrained refinement
*S* = 1.04	*w* = 1/[σ^2^(*F*_o_^2^) + (0.0623*P*)^2^] where *P* = (*F*_o_^2^ + 2*F*_c_^2^)/3
3528 reflections	(Δ/σ)_max_ < 0.001
262 parameters	Δρ_max_ = 0.50 e Å^−^^3^
6 restraints	Δρ_min_ = −0.51 e Å^−^^3^
0 constraints	

### 4-(4-Nitrophenyl)piperazin-1-ium 4-bromobenzoate dihydrate (I) Special details

**Table:**

Geometry. All esds (except the esd in the dihedral angle between two l.s. planes) are estimated using the full covariance matrix. The cell esds are taken into account individually in the estimation of esds in distances, angles and torsion angles; correlations between esds in cell parameters are only used when they are defined by crystal symmetry. An approximate (isotropic) treatment of cell esds is used for estimating esds involving l.s. planes.

### 4-(4-Nitrophenyl)piperazin-1-ium 4-bromobenzoate dihydrate (I) Fractional atomic coordinates and isotropic or equivalent isotropic displacement parameters (Å^2^)

**Table:**

	*x*	*y*	*z*	*U*_iso_*/*U*_eq_	
O1	0.9273 (3)	0.7891 (3)	0.53814 (19)	0.0873 (8)	
O2	0.6916 (3)	0.6357 (3)	0.57409 (17)	0.0767 (6)	
N1	0.3360 (2)	1.0060 (2)	0.29246 (14)	0.0392 (5)	
N2	0.1306 (3)	1.1025 (2)	0.13987 (16)	0.0433 (5)	
N3	0.7641 (3)	0.7395 (3)	0.53118 (17)	0.0537 (6)	
C1	0.4412 (3)	0.9374 (2)	0.34950 (16)	0.0353 (5)	
C2	0.6297 (3)	0.9791 (3)	0.35426 (19)	0.0462 (6)	
H2	0.685335	1.050743	0.316027	0.055*	
C3	0.7335 (3)	0.9160 (3)	0.4144 (2)	0.0479 (6)	
H3	0.858174	0.947226	0.418128	0.057*	
C4	0.6524 (3)	0.8067 (3)	0.46921 (17)	0.0401 (6)	
C5	0.4678 (3)	0.7621 (3)	0.46618 (18)	0.0460 (6)	
H5	0.414057	0.688671	0.503722	0.055*	
C6	0.3636 (3)	0.8264 (3)	0.40761 (18)	0.0430 (6)	
H6	0.239197	0.796118	0.406198	0.052*	
C7	0.4258 (3)	1.1047 (3)	0.2239 (2)	0.0479 (6)	
H7A	0.454966	1.044787	0.17075	0.058*	
H7B	0.537417	1.169773	0.256358	0.058*	
C8	0.3088 (3)	1.1978 (3)	0.1846 (2)	0.0493 (7)	
H8A	0.29158	1.266385	0.236558	0.059*	
H8B	0.368224	1.256083	0.136469	0.059*	
C9	0.0406 (3)	1.0114 (3)	0.2121 (2)	0.0495 (6)	
H9A	−0.074749	0.948618	0.182599	0.059*	
H9B	0.01919	1.076165	0.265103	0.059*	
C10	0.1561 (3)	0.9148 (3)	0.2506 (2)	0.0462 (6)	
H10A	0.097528	0.858526	0.29974	0.055*	
H10B	0.168666	0.844357	0.1984	0.055*	
Br1	0.91659 (5)	0.45794 (4)	0.33416 (3)	0.08360 (18)	
O3	0.4483 (2)	0.78822 (19)	0.01696 (15)	0.0543 (5)	
O4	0.2197 (2)	0.63974 (18)	0.07787 (14)	0.0505 (5)	
C11	0.5153 (3)	0.6356 (2)	0.13620 (18)	0.0350 (5)	
C12	0.6851 (3)	0.6376 (3)	0.10986 (19)	0.0431 (6)	
H12	0.71881	0.674975	0.052451	0.052*	
C13	0.8046 (3)	0.5846 (3)	0.1680 (2)	0.0499 (7)	
H13	0.916747	0.583844	0.149142	0.06*	
C14	0.7551 (3)	0.5332 (3)	0.2538 (2)	0.0466 (6)	
C15	0.5891 (3)	0.5325 (3)	0.28271 (19)	0.0459 (6)	
H15	0.55828	0.499101	0.341588	0.055*	
C16	0.4690 (3)	0.5821 (2)	0.22315 (19)	0.0424 (6)	
H16	0.35572	0.579557	0.241532	0.051*	
C17	0.3848 (3)	0.6919 (2)	0.07250 (19)	0.0391 (6)	
O5	0.7579 (2)	0.01738 (19)	0.02770 (14)	0.0468 (4)	
O6	0.0305 (3)	0.3352 (2)	0.06917 (16)	0.0581 (5)	
H21	0.143 (4)	1.051 (3)	0.0892 (17)	0.07*	
H22	0.075 (4)	1.160 (3)	0.118 (2)	0.07*	
H1W	0.674 (3)	−0.053 (3)	0.031 (2)	0.07*	
H2W	0.720 (4)	0.088 (3)	0.018 (2)	0.07*	
H4W	0.095 (4)	0.421 (2)	0.080 (2)	0.07*	
H3W	−0.041 (4)	0.331 (3)	0.0257 (18)	0.07*	

### 4-(4-Nitrophenyl)piperazin-1-ium 4-bromobenzoate dihydrate (I) Atomic displacement parameters (Å^2^)

**Table:**

	*U*^11^	*U*^22^	*U*^33^	*U*^12^	*U*^13^	*U*^23^
O1	0.0490 (13)	0.0892 (16)	0.122 (2)	0.0142 (11)	−0.0189 (13)	0.0497 (15)
O2	0.0701 (14)	0.0858 (15)	0.0809 (16)	0.0235 (12)	−0.0004 (12)	0.0500 (13)
N1	0.0347 (10)	0.0406 (11)	0.0417 (11)	0.0091 (8)	−0.0042 (9)	0.0124 (9)
N2	0.0459 (12)	0.0431 (12)	0.0435 (13)	0.0189 (10)	−0.0065 (10)	0.0088 (10)
N3	0.0542 (15)	0.0524 (13)	0.0551 (14)	0.0173 (11)	−0.0090 (12)	0.0161 (11)
C1	0.0396 (13)	0.0344 (12)	0.0327 (13)	0.0132 (10)	−0.0010 (10)	0.0026 (10)
C2	0.0378 (14)	0.0518 (15)	0.0515 (16)	0.0111 (11)	0.0047 (12)	0.0229 (12)
C3	0.0361 (13)	0.0503 (15)	0.0583 (17)	0.0125 (11)	−0.0001 (12)	0.0145 (13)
C4	0.0441 (14)	0.0411 (13)	0.0363 (13)	0.0156 (11)	−0.0037 (11)	0.0060 (11)
C5	0.0489 (15)	0.0463 (14)	0.0429 (15)	0.0100 (11)	0.0016 (12)	0.0177 (11)
C6	0.0339 (12)	0.0498 (14)	0.0455 (15)	0.0093 (11)	0.0025 (11)	0.0145 (12)
C7	0.0416 (14)	0.0450 (14)	0.0524 (16)	0.0036 (11)	−0.0082 (12)	0.0166 (12)
C8	0.0558 (16)	0.0385 (13)	0.0499 (16)	0.0096 (12)	−0.0116 (13)	0.0117 (11)
C9	0.0392 (14)	0.0603 (16)	0.0517 (16)	0.0187 (12)	−0.0040 (12)	0.0138 (13)
C10	0.0354 (13)	0.0487 (14)	0.0530 (16)	0.0077 (11)	−0.0030 (12)	0.0159 (12)
Br1	0.0734 (3)	0.0942 (3)	0.0856 (3)	0.0319 (2)	−0.02375 (19)	0.0272 (2)
O3	0.0418 (10)	0.0434 (10)	0.0808 (13)	0.0119 (8)	0.0005 (9)	0.0298 (9)
O4	0.0291 (9)	0.0497 (10)	0.0727 (13)	0.0090 (7)	0.0003 (8)	0.0169 (9)
C11	0.0330 (12)	0.0242 (11)	0.0468 (14)	0.0078 (9)	−0.0007 (11)	0.0021 (10)
C12	0.0403 (14)	0.0432 (13)	0.0504 (16)	0.0160 (11)	0.0087 (12)	0.0125 (11)
C13	0.0345 (13)	0.0548 (15)	0.0659 (19)	0.0194 (11)	0.0057 (13)	0.0145 (14)
C14	0.0443 (15)	0.0381 (13)	0.0548 (17)	0.0096 (11)	−0.0109 (13)	0.0102 (12)
C15	0.0500 (16)	0.0433 (14)	0.0403 (15)	0.0053 (11)	−0.0007 (12)	0.0082 (11)
C16	0.0376 (13)	0.0378 (13)	0.0499 (16)	0.0068 (10)	0.0051 (12)	0.0034 (11)
C17	0.0375 (13)	0.0266 (11)	0.0533 (16)	0.0108 (10)	−0.0010 (11)	0.0032 (11)
O5	0.0370 (10)	0.0413 (10)	0.0619 (12)	0.0103 (7)	−0.0023 (9)	0.0133 (9)
O6	0.0491 (12)	0.0429 (10)	0.0816 (15)	0.0141 (8)	−0.0142 (10)	0.0195 (10)

### 4-(4-Nitrophenyl)piperazin-1-ium 4-bromobenzoate dihydrate (I) Geometric parameters (Å, º)

**Table:**

O1—N3	1.222 (3)	C8—H8B	0.97
O2—N3	1.217 (3)	C9—C10	1.516 (3)
N1—C1	1.391 (3)	C9—H9A	0.97
N1—C7	1.474 (3)	C9—H9B	0.97
N1—C10	1.477 (3)	C10—H10A	0.97
N2—C9	1.476 (4)	C10—H10B	0.97
N2—C8	1.490 (3)	Br1—C14	1.906 (2)
N2—H21	0.850 (17)	O3—C17	1.264 (3)
N2—H22	0.833 (18)	O4—C17	1.257 (3)
N3—C4	1.454 (3)	C11—C16	1.388 (3)
C1—C2	1.405 (3)	C11—C12	1.391 (3)
C1—C6	1.410 (3)	C11—C17	1.506 (3)
C2—C3	1.376 (3)	C12—C13	1.385 (3)
C2—H2	0.93	C12—H12	0.93
C3—C4	1.377 (4)	C13—C14	1.374 (4)
C3—H3	0.93	C13—H13	0.93
C4—C5	1.378 (4)	C14—C15	1.378 (4)
C5—C6	1.372 (3)	C15—C16	1.382 (3)
C5—H5	0.93	C15—H15	0.93
C6—H6	0.93	C16—H16	0.93
C7—C8	1.502 (3)	O5—H1W	0.802 (17)
C7—H7A	0.97	O5—H2W	0.802 (17)
C7—H7B	0.97	O6—H4W	0.823 (17)
C8—H8A	0.97	O6—H3W	0.778 (18)
			
C1—N1—C7	117.46 (19)	N2—C8—H8B	109.4
C1—N1—C10	117.30 (18)	C7—C8—H8B	109.4
C7—N1—C10	112.08 (19)	H8A—C8—H8B	108
C9—N2—C8	109.8 (2)	N2—C9—C10	110.3 (2)
C9—N2—H21	113 (2)	N2—C9—H9A	109.6
C8—N2—H21	110 (2)	C10—C9—H9A	109.6
C9—N2—H22	115 (2)	N2—C9—H9B	109.6
C8—N2—H22	106 (2)	C10—C9—H9B	109.6
H21—N2—H22	102 (3)	H9A—C9—H9B	108.1
O2—N3—O1	122.4 (2)	N1—C10—C9	111.3 (2)
O2—N3—C4	118.8 (2)	N1—C10—H10A	109.4
O1—N3—C4	118.8 (2)	C9—C10—H10A	109.4
N1—C1—C2	121.5 (2)	N1—C10—H10B	109.4
N1—C1—C6	121.4 (2)	C9—C10—H10B	109.4
C2—C1—C6	117.1 (2)	H10A—C10—H10B	108
C3—C2—C1	121.2 (2)	C16—C11—C12	118.6 (2)
C3—C2—H2	119.4	C16—C11—C17	120.5 (2)
C1—C2—H2	119.4	C12—C11—C17	120.9 (2)
C2—C3—C4	119.9 (2)	C13—C12—C11	120.9 (2)
C2—C3—H3	120	C13—C12—H12	119.5
C4—C3—H3	120	C11—C12—H12	119.5
C3—C4—C5	120.6 (2)	C14—C13—C12	119.0 (2)
C3—C4—N3	119.2 (2)	C14—C13—H13	120.5
C5—C4—N3	120.2 (2)	C12—C13—H13	120.5
C6—C5—C4	119.8 (2)	C13—C14—C15	121.4 (2)
C6—C5—H5	120.1	C13—C14—Br1	119.7 (2)
C4—C5—H5	120.1	C15—C14—Br1	118.9 (2)
C5—C6—C1	121.4 (2)	C14—C15—C16	119.2 (2)
C5—C6—H6	119.3	C14—C15—H15	120.4
C1—C6—H6	119.3	C16—C15—H15	120.4
N1—C7—C8	111.5 (2)	C15—C16—C11	120.9 (2)
N1—C7—H7A	109.3	C15—C16—H16	119.6
C8—C7—H7A	109.3	C11—C16—H16	119.6
N1—C7—H7B	109.3	O4—C17—O3	124.3 (2)
C8—C7—H7B	109.3	O4—C17—C11	117.8 (2)
H7A—C7—H7B	108	O3—C17—C11	117.9 (2)
N2—C8—C7	111.17 (19)	H1W—O5—H2W	108 (3)
N2—C8—H8A	109.4	H4W—O6—H3W	109 (3)
C7—C8—H8A	109.4		
			
C7—N1—C1—C2	10.7 (3)	C9—N2—C8—C7	58.1 (3)
C10—N1—C1—C2	148.9 (2)	N1—C7—C8—N2	−55.1 (3)
C7—N1—C1—C6	−171.5 (2)	C8—N2—C9—C10	−58.5 (3)
C10—N1—C1—C6	−33.3 (3)	C1—N1—C10—C9	165.8 (2)
N1—C1—C2—C3	176.8 (2)	C7—N1—C10—C9	−53.9 (3)
C6—C1—C2—C3	−1.1 (4)	N2—C9—C10—N1	56.8 (3)
C1—C2—C3—C4	2.0 (4)	C16—C11—C12—C13	1.3 (3)
C2—C3—C4—C5	−1.6 (4)	C17—C11—C12—C13	−179.4 (2)
C2—C3—C4—N3	179.0 (2)	C11—C12—C13—C14	−1.7 (4)
O2—N3—C4—C3	−174.9 (3)	C12—C13—C14—C15	0.4 (4)
O1—N3—C4—C3	5.0 (4)	C12—C13—C14—Br1	179.36 (19)
O2—N3—C4—C5	5.7 (4)	C13—C14—C15—C16	1.2 (4)
O1—N3—C4—C5	−174.4 (3)	Br1—C14—C15—C16	−177.75 (18)
C3—C4—C5—C6	0.5 (4)	C14—C15—C16—C11	−1.6 (3)
N3—C4—C5—C6	179.9 (2)	C12—C11—C16—C15	0.4 (3)
C4—C5—C6—C1	0.3 (4)	C17—C11—C16—C15	−178.9 (2)
N1—C1—C6—C5	−177.9 (2)	C16—C11—C17—O4	−26.9 (3)
C2—C1—C6—C5	0.0 (4)	C12—C11—C17—O4	153.8 (2)
C1—N1—C7—C8	−166.7 (2)	C16—C11—C17—O3	153.3 (2)
C10—N1—C7—C8	53.0 (3)	C12—C11—C17—O3	−26.0 (3)

### 4-(4-Nitrophenyl)piperazin-1-ium 4-bromobenzoate dihydrate (I) Hydrogen-bond geometry (Å, º)

**Table:**

*D*—H···*A*	*D*—H	H···*A*	*D*···*A*	*D*—H···*A*
N2—H21···O5^i^	0.85 (2)	1.99 (2)	2.810 (3)	162 (3)
N2—H22···O6^ii^	0.83 (2)	1.91 (2)	2.707 (3)	160 (3)
C3—H3···O1^iii^	0.93	2.59	3.260 (4)	130
C13—H13···O4^iv^	0.93	2.57	3.483 (3)	166
C15—H15···O2^v^	0.93	2.47	3.269 (4)	144
O5—H1*W*···O3^vi^	0.80 (2)	1.97 (2)	2.759 (2)	169 (3)
O5—H2*W*···O3^i^	0.80 (2)	2.00 (2)	2.772 (2)	161 (3)
O6—H4*W*···O4	0.82 (2)	2.03 (2)	2.832 (3)	166 (3)
O6—H3*W*···O4^vii^	0.78 (2)	1.99 (2)	2.760 (3)	169 (3)

Symmetry codes: (i) −*x*+1, −*y*+1, −*z*; (ii) *x*, *y*+1, *z*; (iii) −*x*+2, −*y*+2, −*z*+1; (iv) *x*+1, *y*, *z*; (v) −*x*+1, −*y*+1, −*z*+1; (vi) *x*, *y*−1, *z*; (vii) −*x*, −*y*+1, −*z*.

### 4-(4-Nitrophenyl)piperazin-1-ium 4-iodobenzoate dihydrate (II) Crystal data

**Table:**

C_10_H_14_N_3_O_2_^+^·C_7_H_4_IO_2_^−^·2H_2_O	*Z* = 2
*M**_r_* = 491.28	*F*(000) = 492
Triclinic, *P*1	*D*_x_ = 1.699 Mg m^−^^3^
Hall symbol: -P 1	Mo *K*α radiation, λ = 0.71073 Å
*a* = 7.7652 (4) Å	Cell parameters from 6331 reflections
*b* = 9.2852 (5) Å	θ = 2.6–25.4°
*c* = 13.930 (1) Å	µ = 1.71 mm^−^^1^
α = 94.985 (5)°	*T* = 293 K
β = 95.331 (5)°	Prism, brown
γ = 104.875 (6)°	0.48 × 0.48 × 0.2 mm
*V* = 960.09 (10) Å^3^	

### 4-(4-Nitrophenyl)piperazin-1-ium 4-iodobenzoate dihydrate (II) Data collection

**Table:**

Oxford Diffraction Xcalibur diffractometer	2952 reflections with *I* > 2σ(*I*)
ω scans	*R*_int_ = 0.017
Absorption correction: multi-scan (CrysAlis RED; Oxford Diffraction, 2009)	θ_max_ = 25.4°, θ_min_ = 2.6°
*T*_min_ = 0.458, *T*_max_ = 0.711	*h* = −8→9
6331 measured reflections	*k* = −11→9
3518 independent reflections	*l* = −14→16

### 4-(4-Nitrophenyl)piperazin-1-ium 4-iodobenzoate dihydrate (II) Refinement

**Table:**

Refinement on *F*^2^	0 constraints
Least-squares matrix: full	Hydrogen site location: mixed
*R*[*F*^2^ > 2σ(*F*^2^)] = 0.029	H atoms treated by a mixture of independent and constrained refinement
*wR*(*F*^2^) = 0.069	*w* = 1/[σ^2^(*F*_o_^2^) + (0.0319*P*)^2^ + 0.5892*P*] where *P* = (*F*_o_^2^ + 2*F*_c_^2^)/3
*S* = 1.03	(Δ/σ)_max_ < 0.001
3513 reflections	Δρ_max_ = 0.54 e Å^−^^3^
262 parameters	Δρ_min_ = −0.66 e Å^−^^3^
6 restraints	

### 4-(4-Nitrophenyl)piperazin-1-ium 4-iodobenzoate dihydrate (II) Special details

**Table:**

Geometry. All esds (except the esd in the dihedral angle between two l.s. planes) are estimated using the full covariance matrix. The cell esds are taken into account individually in the estimation of esds in distances, angles and torsion angles; correlations between esds in cell parameters are only used when they are defined by crystal symmetry. An approximate (isotropic) treatment of cell esds is used for estimating esds involving l.s. planes.

### 4-(4-Nitrophenyl)piperazin-1-ium 4-iodobenzoate dihydrate (II) Fractional atomic coordinates and isotropic or equivalent isotropic displacement parameters (Å^2^)

**Table:**

	*x*	*y*	*z*	*U*_iso_*/*U*_eq_	
O1	0.9211 (3)	0.7945 (3)	0.5327 (2)	0.0765 (8)	
O2	0.6869 (3)	0.6415 (3)	0.5706 (2)	0.0702 (7)	
N1	0.3324 (3)	1.0055 (2)	0.29012 (17)	0.0344 (5)	
N2	0.1277 (3)	1.1016 (3)	0.13972 (19)	0.0404 (6)	
N3	0.7583 (4)	0.7448 (3)	0.52680 (19)	0.0471 (6)	
C1	0.4369 (3)	0.9384 (3)	0.34660 (19)	0.0311 (6)	
C2	0.6239 (4)	0.9794 (3)	0.3495 (2)	0.0421 (7)	
H2	0.678241	1.049839	0.310458	0.051*	
C3	0.7273 (4)	0.9175 (3)	0.4087 (2)	0.0434 (7)	
H3	0.851637	0.947138	0.411003	0.052*	
C4	0.6475 (4)	0.8107 (3)	0.4653 (2)	0.0348 (6)	
C5	0.4638 (4)	0.7665 (3)	0.4644 (2)	0.0407 (7)	
H5	0.411197	0.694461	0.502907	0.049*	
C6	0.3609 (4)	0.8297 (3)	0.4063 (2)	0.0390 (7)	
H6	0.236893	0.800552	0.405831	0.047*	
C7	0.4203 (4)	1.1048 (3)	0.2222 (2)	0.0412 (7)	
H7A	0.448427	1.044706	0.168453	0.049*	
H7B	0.532139	1.170403	0.255077	0.049*	
C8	0.3038 (4)	1.1984 (3)	0.1836 (2)	0.0439 (7)	
H8A	0.287569	1.267775	0.236	0.053*	
H8B	0.361796	1.256375	0.135285	0.053*	
C9	0.0381 (4)	1.0095 (4)	0.2115 (2)	0.0456 (7)	
H9A	−0.07676	0.945606	0.181239	0.055*	
H9B	0.016429	1.074515	0.264773	0.055*	
C10	0.1531 (4)	0.9144 (3)	0.2497 (2)	0.0413 (7)	
H10A	0.096206	0.859686	0.299686	0.05*	
H10B	0.163241	0.841846	0.19758	0.05*	
I1	0.92211 (3)	0.44728 (3)	0.33815 (2)	0.05798 (10)	
O3	0.4459 (3)	0.7894 (2)	0.01595 (18)	0.0503 (6)	
O4	0.2183 (3)	0.6411 (2)	0.07685 (16)	0.0463 (5)	
C11	0.5106 (3)	0.6368 (3)	0.1340 (2)	0.0321 (6)	
C12	0.6798 (4)	0.6390 (3)	0.1072 (2)	0.0387 (7)	
H12	0.71341	0.676998	0.050132	0.046*	
C13	0.7975 (4)	0.5851 (3)	0.1648 (2)	0.0411 (7)	
H13	0.909115	0.584052	0.146196	0.049*	
C14	0.7470 (4)	0.5326 (3)	0.2509 (2)	0.0376 (7)	
C15	0.5813 (4)	0.5331 (3)	0.2798 (2)	0.0398 (7)	
H15	0.550434	0.499714	0.338523	0.048*	
C16	0.4631 (4)	0.5834 (3)	0.2208 (2)	0.0378 (7)	
H16	0.350345	0.581695	0.238875	0.045*	
C17	0.3825 (4)	0.6931 (3)	0.0705 (2)	0.0356 (6)	
O5	0.7577 (3)	0.0174 (2)	0.02906 (17)	0.0446 (5)	
O6	0.0304 (3)	0.3341 (3)	0.06807 (19)	0.0529 (6)	
H21	0.144 (5)	1.051 (4)	0.0882 (19)	0.063*	
H22	0.076 (4)	1.162 (3)	0.116 (3)	0.063*	
H1W	0.671 (4)	−0.051 (3)	0.032 (3)	0.063*	
H2W	0.711 (5)	0.084 (3)	0.022 (3)	0.063*	
H4W	0.100 (4)	0.414 (3)	0.080 (3)	0.063*	
H3W	−0.052 (4)	0.326 (4)	0.027 (2)	0.063*	

### 4-(4-Nitrophenyl)piperazin-1-ium 4-iodobenzoate dihydrate (II) Atomic displacement parameters (Å^2^)

**Table:**

	*U*^11^	*U*^22^	*U*^33^	*U*^12^	*U*^13^	*U*^23^
O1	0.0437 (15)	0.0752 (18)	0.108 (2)	0.0122 (13)	−0.0192 (14)	0.0400 (16)
O2	0.0633 (16)	0.0754 (18)	0.0786 (18)	0.0200 (13)	0.0033 (13)	0.0469 (15)
N1	0.0300 (12)	0.0321 (13)	0.0391 (13)	0.0054 (10)	−0.0037 (10)	0.0090 (10)
N2	0.0420 (14)	0.0388 (15)	0.0413 (15)	0.0159 (11)	−0.0066 (12)	0.0072 (11)
N3	0.0496 (17)	0.0450 (16)	0.0461 (15)	0.0151 (13)	−0.0076 (13)	0.0084 (13)
C1	0.0328 (14)	0.0280 (14)	0.0316 (14)	0.0093 (11)	−0.0008 (11)	0.0009 (11)
C2	0.0340 (15)	0.0433 (17)	0.0496 (18)	0.0067 (13)	0.0047 (13)	0.0196 (14)
C3	0.0279 (15)	0.0464 (18)	0.0548 (19)	0.0086 (13)	−0.0010 (13)	0.0115 (15)
C4	0.0369 (15)	0.0344 (15)	0.0332 (15)	0.0118 (12)	−0.0029 (12)	0.0049 (12)
C5	0.0422 (17)	0.0417 (17)	0.0383 (16)	0.0087 (13)	0.0047 (13)	0.0139 (13)
C6	0.0292 (14)	0.0443 (17)	0.0430 (17)	0.0073 (12)	0.0016 (12)	0.0118 (13)
C7	0.0353 (15)	0.0389 (17)	0.0459 (17)	0.0034 (13)	−0.0040 (13)	0.0148 (14)
C8	0.0483 (18)	0.0350 (16)	0.0447 (17)	0.0078 (14)	−0.0075 (14)	0.0095 (13)
C9	0.0354 (16)	0.055 (2)	0.0479 (18)	0.0160 (14)	−0.0023 (14)	0.0103 (15)
C10	0.0297 (15)	0.0401 (17)	0.0524 (18)	0.0071 (13)	−0.0035 (13)	0.0110 (14)
I1	0.05063 (15)	0.06217 (17)	0.06139 (16)	0.01852 (11)	−0.01269 (10)	0.01870 (11)
O3	0.0370 (11)	0.0400 (12)	0.0758 (16)	0.0088 (9)	0.0016 (11)	0.0276 (11)
O4	0.0283 (11)	0.0460 (12)	0.0629 (14)	0.0073 (9)	−0.0016 (10)	0.0134 (10)
C11	0.0290 (14)	0.0241 (14)	0.0415 (16)	0.0059 (11)	−0.0004 (12)	0.0023 (12)
C12	0.0376 (16)	0.0394 (16)	0.0431 (17)	0.0138 (13)	0.0088 (13)	0.0119 (13)
C13	0.0315 (15)	0.0436 (17)	0.0516 (19)	0.0157 (13)	0.0044 (13)	0.0082 (14)
C14	0.0336 (15)	0.0306 (15)	0.0458 (17)	0.0078 (12)	−0.0064 (13)	0.0047 (13)
C15	0.0421 (17)	0.0365 (16)	0.0373 (16)	0.0043 (13)	0.0025 (13)	0.0068 (13)
C16	0.0288 (14)	0.0356 (16)	0.0471 (17)	0.0062 (12)	0.0048 (13)	0.0018 (13)
C17	0.0328 (15)	0.0241 (14)	0.0475 (17)	0.0073 (12)	−0.0011 (13)	−0.0012 (12)
O5	0.0345 (11)	0.0374 (13)	0.0610 (14)	0.0094 (9)	−0.0027 (10)	0.0111 (11)
O6	0.0442 (13)	0.0383 (12)	0.0735 (17)	0.0097 (10)	−0.0123 (11)	0.0164 (12)

### 4-(4-Nitrophenyl)piperazin-1-ium 4-iodobenzoate dihydrate (II) Geometric parameters (Å, º)

**Table:**

O1—N3	1.222 (3)	C8—H8B	0.97
O2—N3	1.221 (3)	C9—C10	1.503 (4)
N1—C1	1.376 (3)	C9—H9A	0.97
N1—C10	1.462 (3)	C9—H9B	0.97
N1—C7	1.469 (4)	C10—H10A	0.97
N2—C8	1.473 (4)	C10—H10B	0.97
N2—C9	1.479 (4)	I1—C14	2.090 (3)
N2—H21	0.859 (18)	O3—C17	1.257 (3)
N2—H22	0.850 (18)	O4—C17	1.257 (3)
N3—C4	1.440 (4)	C11—C16	1.391 (4)
C1—C2	1.400 (4)	C11—C12	1.395 (4)
C1—C6	1.410 (4)	C11—C17	1.493 (4)
C2—C3	1.361 (4)	C12—C13	1.377 (4)
C2—H2	0.93	C12—H12	0.93
C3—C4	1.378 (4)	C13—C14	1.386 (4)
C3—H3	0.93	C13—H13	0.93
C4—C5	1.378 (4)	C14—C15	1.385 (4)
C5—C6	1.357 (4)	C15—C16	1.372 (4)
C5—H5	0.93	C15—H15	0.93
C6—H6	0.93	C16—H16	0.93
C7—C8	1.502 (4)	O5—H1W	0.805 (18)
C7—H7A	0.97	O5—H2W	0.805 (18)
C7—H7B	0.97	O6—H4W	0.795 (18)
C8—H8A	0.97	O6—H3W	0.800 (18)
			
C1—N1—C10	117.2 (2)	N2—C8—H8B	109.6
C1—N1—C7	117.8 (2)	C7—C8—H8B	109.6
C10—N1—C7	112.8 (2)	H8A—C8—H8B	108.1
C8—N2—C9	110.6 (2)	N2—C9—C10	110.3 (2)
C8—N2—H21	108 (3)	N2—C9—H9A	109.6
C9—N2—H21	115 (3)	C10—C9—H9A	109.6
C8—N2—H22	104 (3)	N2—C9—H9B	109.6
C9—N2—H22	118 (3)	C10—C9—H9B	109.6
H21—N2—H22	101 (3)	H9A—C9—H9B	108.1
O2—N3—O1	122.5 (3)	N1—C10—C9	111.5 (2)
O2—N3—C4	119.1 (3)	N1—C10—H10A	109.3
O1—N3—C4	118.4 (3)	C9—C10—H10A	109.3
N1—C1—C2	121.1 (2)	N1—C10—H10B	109.3
N1—C1—C6	121.5 (2)	C9—C10—H10B	109.3
C2—C1—C6	117.4 (2)	H10A—C10—H10B	108
C3—C2—C1	120.9 (3)	C16—C11—C12	119.5 (3)
C3—C2—H2	119.6	C16—C11—C17	120.4 (2)
C1—C2—H2	119.6	C12—C11—C17	120.1 (2)
C2—C3—C4	119.9 (3)	C13—C12—C11	120.4 (3)
C2—C3—H3	120.1	C13—C12—H12	119.8
C4—C3—H3	120.1	C11—C12—H12	119.8
C5—C4—C3	121.2 (3)	C12—C13—C14	119.0 (3)
C5—C4—N3	119.5 (3)	C12—C13—H13	120.5
C3—C4—N3	119.3 (3)	C14—C13—H13	120.5
C6—C5—C4	118.9 (3)	C15—C14—C13	121.4 (3)
C6—C5—H5	120.5	C15—C14—I1	119.1 (2)
C4—C5—H5	120.5	C13—C14—I1	119.6 (2)
C5—C6—C1	121.8 (3)	C16—C15—C14	119.2 (3)
C5—C6—H6	119.1	C16—C15—H15	120.4
C1—C6—H6	119.1	C14—C15—H15	120.4
N1—C7—C8	111.9 (2)	C15—C16—C11	120.5 (3)
N1—C7—H7A	109.2	C15—C16—H16	119.8
C8—C7—H7A	109.2	C11—C16—H16	119.8
N1—C7—H7B	109.2	O4—C17—O3	125.0 (3)
C8—C7—H7B	109.2	O4—C17—C11	116.9 (2)
H7A—C7—H7B	107.9	O3—C17—C11	118.1 (2)
N2—C8—C7	110.2 (2)	H1W—O5—H2W	101 (4)
N2—C8—H8A	109.6	H4W—O6—H3W	117 (4)
C7—C8—H8A	109.6		
			
C10—N1—C1—C2	148.6 (3)	C9—N2—C8—C7	58.1 (3)
C7—N1—C1—C2	8.9 (4)	N1—C7—C8—N2	−54.7 (3)
C10—N1—C1—C6	−33.7 (4)	C8—N2—C9—C10	−58.5 (3)
C7—N1—C1—C6	−173.4 (3)	C1—N1—C10—C9	165.8 (3)
N1—C1—C2—C3	177.1 (3)	C7—N1—C10—C9	−52.6 (3)
C6—C1—C2—C3	−0.8 (4)	N2—C9—C10—N1	55.3 (4)
C1—C2—C3—C4	1.3 (5)	C16—C11—C12—C13	1.6 (4)
C2—C3—C4—C5	−1.0 (5)	C17—C11—C12—C13	−179.0 (3)
C2—C3—C4—N3	179.3 (3)	C11—C12—C13—C14	−1.7 (4)
O2—N3—C4—C5	6.3 (4)	C12—C13—C14—C15	0.1 (4)
O1—N3—C4—C5	−173.9 (3)	C12—C13—C14—I1	179.0 (2)
O2—N3—C4—C3	−173.9 (3)	C13—C14—C15—C16	1.6 (4)
O1—N3—C4—C3	5.8 (4)	I1—C14—C15—C16	−177.4 (2)
C3—C4—C5—C6	0.1 (5)	C14—C15—C16—C11	−1.6 (4)
N3—C4—C5—C6	179.8 (3)	C12—C11—C16—C15	0.0 (4)
C4—C5—C6—C1	0.4 (5)	C17—C11—C16—C15	−179.3 (3)
N1—C1—C6—C5	−178.0 (3)	C16—C11—C17—O4	−26.0 (4)
C2—C1—C6—C5	−0.1 (4)	C12—C11—C17—O4	154.6 (3)
C1—N1—C7—C8	−166.2 (2)	C16—C11—C17—O3	153.1 (3)
C10—N1—C7—C8	52.5 (3)	C12—C11—C17—O3	−26.2 (4)

### 4-(4-Nitrophenyl)piperazin-1-ium 4-iodobenzoate dihydrate (II) Hydrogen-bond geometry (Å, º)

**Table:**

*D*—H···*A*	*D*—H	H···*A*	*D*···*A*	*D*—H···*A*
N2—H21···O5^i^	0.86 (2)	1.99 (2)	2.825 (4)	164 (4)
N2—H22···O6^ii^	0.85 (2)	1.88 (2)	2.702 (3)	163 (4)
C3—H3···O1^iii^	0.93	2.59	3.275 (4)	131
C13—H13···O4^iv^	0.93	2.62	3.526 (4)	166
C15—H15···O2^v^	0.93	2.49	3.311 (4)	147
O5—H1*W*···O3^vi^	0.81 (2)	1.96 (2)	2.756 (3)	170 (4)
O5—H2*W*···O3^i^	0.81 (2)	1.96 (2)	2.753 (3)	166 (4)
O6—H4*W*···O4	0.80 (2)	2.08 (2)	2.836 (3)	160 (4)
O6—H3*W*···O4^vii^	0.80 (2)	1.95 (2)	2.728 (3)	165 (4)

Symmetry codes: (i) −*x*+1, −*y*+1, −*z*; (ii) *x*, *y*+1, *z*; (iii) −*x*+2, −*y*+2, −*z*+1; (iv) *x*+1, *y*, *z*; (v) −*x*+1, −*y*+1, −*z*+1; (vi) *x*, *y*−1, *z*; (vii) −*x*, −*y*+1, −*z*.

### 4-(4-Nitrophenyl)piperazin-1-ium 4-hydroxybenzoate monohydrate (III) Crystal data

**Table:**

C_10_H_14_N_3_O_2_^+^·C_7_H_5_O_3_^−^·H_2_O	*Z* = 2
*M**_r_* = 363.37	*F*(000) = 384
Triclinic, *P*1	*D*_x_ = 1.407 Mg m^−^^3^
Hall symbol: -P 1	Mo *K*α radiation, λ = 0.71073 Å
*a* = 9.636 (1) Å	Cell parameters from 5342 reflections
*b* = 10.301 (1) Å	θ = 2.6–25.3°
*c* = 10.867 (1) Å	µ = 0.11 mm^−^^1^
α = 103.90 (1)°	*T* = 293 K
β = 108.32 (1)°	Rod, yellow
γ = 112.96 (1)°	0.50 × 0.32 × 0.24 mm
*V* = 857.80 (17) Å^3^	

### 4-(4-Nitrophenyl)piperazin-1-ium 4-hydroxybenzoate monohydrate (III) Data collection

**Table:**

Oxford Diffraction Xcalibur diffractometer	2342 reflections with *I* > 2σ(*I*)
ω scans	*R*_int_ = 0.013
Absorption correction: multi-scan (CrysAlis RED; Oxford Diffraction, 2009)	θ_max_ = 25.3°, θ_min_ = 2.6°
*T*_min_ = 0.959, *T*_max_ = 0.974	*h* = −11→11
5342 measured reflections	*k* = −12→11
3140 independent reflections	*l* = −13→12

### 4-(4-Nitrophenyl)piperazin-1-ium 4-hydroxybenzoate monohydrate (III) Refinement

**Table:**

Refinement on *F*^2^	Hydrogen site location: mixed
Least-squares matrix: full	H atoms treated by a mixture of independent and constrained refinement
*R*[*F*^2^ > 2σ(*F*^2^)] = 0.043	*w* = 1/[σ^2^(*F*_o_^2^) + (0.0386*P*)^2^ + 0.3231*P*] where *P* = (*F*_o_^2^ + 2*F*_c_^2^)/3
*wR*(*F*^2^) = 0.106	(Δ/σ)_max_ < 0.001
*S* = 1.05	Δρ_max_ = 0.19 e Å^−^^3^
3135 reflections	Δρ_min_ = −0.19 e Å^−^^3^
251 parameters	Extinction correction: SHELXL2018/3 (Sheldrick 2015b), Fc^*^=kFc[1+0.001xFc^2^λ^3^/sin(2θ)]^-1/4^
5 restraints	Extinction coefficient: 0.032 (3)
0 constraints	

### 4-(4-Nitrophenyl)piperazin-1-ium 4-hydroxybenzoate monohydrate (III) Special details

**Table:**

Geometry. All esds (except the esd in the dihedral angle between two l.s. planes) are estimated using the full covariance matrix. The cell esds are taken into account individually in the estimation of esds in distances, angles and torsion angles; correlations between esds in cell parameters are only used when they are defined by crystal symmetry. An approximate (isotropic) treatment of cell esds is used for estimating esds involving l.s. planes.

### 4-(4-Nitrophenyl)piperazin-1-ium 4-hydroxybenzoate monohydrate (III) Fractional atomic coordinates and isotropic or equivalent isotropic displacement parameters (Å^2^)

**Table:**

	*x*	*y*	*z*	*U*_iso_*/*U*_eq_	
C1	0.3138 (2)	0.37000 (19)	0.65529 (18)	0.0346 (4)	
C2	0.1431 (2)	0.2527 (2)	0.57638 (19)	0.0418 (4)	
H2	0.068235	0.250847	0.613904	0.05*	
C3	0.0843 (2)	0.1407 (2)	0.4450 (2)	0.0434 (5)	
H3	−0.029409	0.063818	0.39416	0.052*	
C4	0.1942 (2)	0.1428 (2)	0.38907 (19)	0.0411 (4)	
C5	0.3624 (2)	0.2553 (2)	0.4636 (2)	0.0483 (5)	
H5	0.436252	0.255393	0.42532	0.058*	
C6	0.4208 (2)	0.3670 (2)	0.5942 (2)	0.0472 (5)	
H6	0.53484	0.443048	0.64379	0.057*	
C7	0.5230 (2)	0.6338 (2)	0.8284 (2)	0.0453 (5)	
H7A	0.489341	0.67826	0.763471	0.054*	
H7B	0.611237	0.616548	0.817407	0.054*	
C8	0.5933 (2)	0.7472 (2)	0.9784 (2)	0.0493 (5)	
H8A	0.643572	0.71093	1.044494	0.059*	
H8B	0.681545	0.846719	0.99504	0.059*	
C9	0.3302 (3)	0.6143 (2)	0.9836 (2)	0.0457 (5)	
H9A	0.242868	0.625614	1.002896	0.055*	
H9B	0.381743	0.579404	1.050389	0.055*	
C10	0.2514 (2)	0.4953 (2)	0.83370 (19)	0.0403 (4)	
H10A	0.176601	0.394834	0.82667	0.048*	
H10B	0.183115	0.521491	0.76885	0.048*	
C11	0.1749 (2)	0.5843 (2)	0.29147 (19)	0.0418 (5)	
C12	0.2469 (3)	0.7413 (2)	0.3258 (2)	0.0495 (5)	
H12	0.262285	0.778209	0.258209	0.059*	
C13	0.2959 (2)	0.8431 (2)	0.4597 (2)	0.0438 (5)	
H13	0.344454	0.94863	0.481898	0.053*	
C14	0.2738 (2)	0.7906 (2)	0.56226 (18)	0.0364 (4)	
C15	0.2004 (2)	0.6329 (2)	0.52544 (19)	0.0395 (4)	
H15	0.18337	0.595519	0.592378	0.047*	
C16	0.1520 (2)	0.5299 (2)	0.39188 (19)	0.0410 (4)	
H16	0.104259	0.424435	0.369591	0.049*	
C17	0.3293 (2)	0.9024 (2)	0.7080 (2)	0.0440 (5)	
N1	0.37669 (18)	0.48418 (16)	0.78933 (15)	0.0372 (4)	
N2	0.4599 (2)	0.76559 (19)	1.00415 (18)	0.0479 (4)	
N3	0.1322 (2)	0.02537 (19)	0.25010 (18)	0.0518 (4)	
O1	0.2282 (2)	0.04014 (19)	0.19537 (17)	0.0759 (5)	
O2	−0.0112 (2)	−0.08579 (18)	0.19233 (16)	0.0715 (5)	
O3	0.1311 (2)	0.48813 (18)	0.15852 (15)	0.0637 (4)	
O4	0.3985 (2)	1.04290 (17)	0.73256 (16)	0.0744 (5)	
O5	0.30323 (18)	0.85085 (16)	0.79750 (14)	0.0547 (4)	
O6	−0.0679 (3)	0.1917 (2)	0.09903 (19)	0.0809 (6)	
H21	0.412 (3)	0.796 (3)	0.941 (2)	0.097*	
H22	0.513 (3)	0.838 (3)	1.099 (2)	0.097*	
H17	0.070 (3)	0.394 (2)	0.143 (3)	0.097*	
H1W	−0.140 (3)	0.168 (3)	0.129 (3)	0.097*	
H2W	−0.112 (3)	0.128 (3)	0.015 (2)	0.097*	

### 4-(4-Nitrophenyl)piperazin-1-ium 4-hydroxybenzoate monohydrate (III) Atomic displacement parameters (Å^2^)

**Table:**

	*U*^11^	*U*^22^	*U*^33^	*U*^12^	*U*^13^	*U*^23^
C1	0.0348 (10)	0.0343 (9)	0.0377 (10)	0.0187 (8)	0.0167 (8)	0.0173 (8)
C2	0.0357 (10)	0.0427 (10)	0.0444 (11)	0.0159 (8)	0.0211 (8)	0.0168 (9)
C3	0.0356 (10)	0.0380 (10)	0.0439 (11)	0.0113 (8)	0.0148 (8)	0.0145 (9)
C4	0.0463 (11)	0.0347 (9)	0.0373 (10)	0.0191 (8)	0.0175 (8)	0.0119 (8)
C5	0.0429 (11)	0.0481 (11)	0.0505 (12)	0.0215 (9)	0.0255 (9)	0.0120 (10)
C6	0.0315 (10)	0.0440 (11)	0.0514 (12)	0.0139 (8)	0.0177 (9)	0.0081 (9)
C7	0.0379 (10)	0.0401 (10)	0.0487 (11)	0.0133 (8)	0.0209 (9)	0.0137 (9)
C8	0.0459 (11)	0.0395 (11)	0.0468 (11)	0.0154 (9)	0.0160 (9)	0.0117 (9)
C9	0.0590 (12)	0.0451 (11)	0.0434 (11)	0.0277 (10)	0.0295 (10)	0.0231 (9)
C10	0.0429 (10)	0.0421 (10)	0.0420 (10)	0.0213 (9)	0.0239 (9)	0.0211 (9)
C11	0.0398 (10)	0.0443 (11)	0.0361 (10)	0.0170 (9)	0.0194 (8)	0.0133 (9)
C12	0.0595 (13)	0.0525 (12)	0.0441 (11)	0.0248 (10)	0.0315 (10)	0.0269 (10)
C13	0.0494 (11)	0.0392 (10)	0.0482 (11)	0.0207 (9)	0.0274 (9)	0.0224 (9)
C14	0.0357 (9)	0.0405 (10)	0.0381 (10)	0.0210 (8)	0.0190 (8)	0.0183 (8)
C15	0.0410 (10)	0.0440 (10)	0.0378 (10)	0.0201 (8)	0.0208 (8)	0.0223 (9)
C16	0.0409 (10)	0.0356 (10)	0.0423 (11)	0.0151 (8)	0.0194 (8)	0.0167 (8)
C17	0.0482 (11)	0.0462 (12)	0.0409 (11)	0.0255 (9)	0.0218 (9)	0.0180 (9)
N1	0.0349 (8)	0.0348 (8)	0.0385 (8)	0.0157 (7)	0.0170 (7)	0.0130 (7)
N2	0.0616 (11)	0.0410 (9)	0.0425 (10)	0.0259 (8)	0.0257 (9)	0.0168 (8)
N3	0.0575 (11)	0.0430 (10)	0.0443 (10)	0.0214 (9)	0.0209 (9)	0.0124 (8)
O1	0.0806 (12)	0.0662 (11)	0.0599 (10)	0.0220 (9)	0.0434 (9)	0.0052 (8)
O2	0.0600 (10)	0.0523 (9)	0.0564 (10)	0.0087 (8)	0.0163 (8)	0.0010 (8)
O3	0.0759 (11)	0.0545 (9)	0.0430 (8)	0.0171 (8)	0.0337 (8)	0.0121 (7)
O4	0.1147 (14)	0.0416 (9)	0.0554 (10)	0.0278 (9)	0.0450 (10)	0.0145 (7)
O5	0.0721 (10)	0.0620 (9)	0.0425 (8)	0.0375 (8)	0.0324 (7)	0.0259 (7)
O6	0.0936 (14)	0.0547 (10)	0.0652 (11)	0.0142 (10)	0.0484 (10)	0.0041 (8)

### 4-(4-Nitrophenyl)piperazin-1-ium 4-hydroxybenzoate monohydrate (III) Geometric parameters (Å, º)

**Table:**

C1—N1	1.392 (2)	C10—H10A	0.97
C1—C6	1.397 (2)	C10—H10B	0.97
C1—C2	1.402 (2)	C11—O3	1.360 (2)
C2—C3	1.371 (3)	C11—C16	1.379 (3)
C2—H2	0.93	C11—C12	1.382 (3)
C3—C4	1.373 (3)	C12—C13	1.374 (3)
C3—H3	0.93	C12—H12	0.93
C4—C5	1.373 (3)	C13—C14	1.389 (2)
C4—N3	1.446 (2)	C13—H13	0.93
C5—C6	1.365 (3)	C14—C15	1.383 (2)
C5—H5	0.93	C14—C17	1.494 (3)
C6—H6	0.93	C15—C16	1.378 (2)
C7—N1	1.468 (2)	C15—H15	0.93
C7—C8	1.501 (3)	C16—H16	0.93
C7—H7A	0.97	C17—O4	1.250 (2)
C7—H7B	0.97	C17—O5	1.260 (2)
C8—N2	1.470 (3)	N2—H21	0.893 (17)
C8—H8A	0.97	N2—H22	0.935 (17)
C8—H8B	0.97	N3—O2	1.221 (2)
C9—N2	1.476 (2)	N3—O1	1.230 (2)
C9—C10	1.507 (3)	O3—H17	0.850 (17)
C9—H9A	0.97	O6—H1W	0.832 (17)
C9—H9B	0.97	O6—H2W	0.834 (17)
C10—N1	1.467 (2)		
			
N1—C1—C6	120.65 (15)	N1—C10—H10B	108.9
N1—C1—C2	122.40 (15)	C9—C10—H10B	108.9
C6—C1—C2	116.95 (16)	H10A—C10—H10B	107.8
C3—C2—C1	121.37 (17)	O3—C11—C16	122.11 (17)
C3—C2—H2	119.3	O3—C11—C12	118.10 (17)
C1—C2—H2	119.3	C16—C11—C12	119.78 (17)
C2—C3—C4	119.60 (17)	C13—C12—C11	120.14 (18)
C2—C3—H3	120.2	C13—C12—H12	119.9
C4—C3—H3	120.2	C11—C12—H12	119.9
C3—C4—C5	120.68 (17)	C12—C13—C14	120.94 (17)
C3—C4—N3	119.73 (17)	C12—C13—H13	119.5
C5—C4—N3	119.59 (17)	C14—C13—H13	119.5
C6—C5—C4	119.67 (17)	C15—C14—C13	118.02 (16)
C6—C5—H5	120.2	C15—C14—C17	121.42 (16)
C4—C5—H5	120.2	C13—C14—C17	120.55 (16)
C5—C6—C1	121.72 (17)	C16—C15—C14	121.51 (17)
C5—C6—H6	119.1	C16—C15—H15	119.2
C1—C6—H6	119.1	C14—C15—H15	119.2
N1—C7—C8	113.07 (16)	C15—C16—C11	119.60 (17)
N1—C7—H7A	109	C15—C16—H16	120.2
C8—C7—H7A	109	C11—C16—H16	120.2
N1—C7—H7B	109	O4—C17—O5	124.14 (18)
C8—C7—H7B	109	O4—C17—C14	116.82 (17)
H7A—C7—H7B	107.8	O5—C17—C14	119.04 (17)
N2—C8—C7	110.93 (16)	C1—N1—C10	116.66 (14)
N2—C8—H8A	109.5	C1—N1—C7	115.79 (14)
C7—C8—H8A	109.5	C10—N1—C7	114.73 (14)
N2—C8—H8B	109.5	C8—N2—C9	108.67 (15)
C7—C8—H8B	109.5	C8—N2—H21	109.1 (18)
H8A—C8—H8B	108	C9—N2—H21	108.9 (18)
N2—C9—C10	110.96 (15)	C8—N2—H22	106.2 (17)
N2—C9—H9A	109.4	C9—N2—H22	110.8 (17)
C10—C9—H9A	109.4	H21—N2—H22	113 (2)
N2—C9—H9B	109.4	O2—N3—O1	122.24 (17)
C10—C9—H9B	109.4	O2—N3—C4	119.43 (17)
H9A—C9—H9B	108	O1—N3—C4	118.32 (17)
N1—C10—C9	113.15 (15)	C11—O3—H17	110.2 (19)
N1—C10—H10A	108.9	H1W—O6—H2W	108 (3)
C9—C10—H10A	108.9		
			
N1—C1—C2—C3	−179.46 (17)	O3—C11—C16—C15	179.34 (17)
C6—C1—C2—C3	−0.3 (3)	C12—C11—C16—C15	0.4 (3)
C1—C2—C3—C4	0.0 (3)	C15—C14—C17—O4	178.09 (18)
C2—C3—C4—C5	0.4 (3)	C13—C14—C17—O4	−1.4 (3)
C2—C3—C4—N3	−179.58 (17)	C15—C14—C17—O5	−2.4 (3)
C3—C4—C5—C6	−0.5 (3)	C13—C14—C17—O5	178.03 (18)
N3—C4—C5—C6	179.41 (18)	C6—C1—N1—C10	169.31 (17)
C4—C5—C6—C1	0.3 (3)	C2—C1—N1—C10	−11.5 (2)
N1—C1—C6—C5	179.30 (18)	C6—C1—N1—C7	29.6 (2)
C2—C1—C6—C5	0.1 (3)	C2—C1—N1—C7	−151.19 (18)
N1—C7—C8—N2	−53.2 (2)	C9—C10—N1—C1	176.12 (15)
N2—C9—C10—N1	52.1 (2)	C9—C10—N1—C7	−43.8 (2)
O3—C11—C12—C13	−178.90 (18)	C8—C7—N1—C1	−175.25 (16)
C16—C11—C12—C13	0.1 (3)	C8—C7—N1—C10	44.3 (2)
C11—C12—C13—C14	−0.2 (3)	C7—C8—N2—C9	61.2 (2)
C12—C13—C14—C15	−0.3 (3)	C10—C9—N2—C8	−60.7 (2)
C12—C13—C14—C17	179.26 (18)	C3—C4—N3—O2	−9.7 (3)
C13—C14—C15—C16	0.8 (3)	C5—C4—N3—O2	170.3 (2)
C17—C14—C15—C16	−178.76 (17)	C3—C4—N3—O1	171.61 (19)
C14—C15—C16—C11	−0.8 (3)	C5—C4—N3—O1	−8.3 (3)

### 4-(4-Nitrophenyl)piperazin-1-ium 4-hydroxybenzoate monohydrate (III) Hydrogen-bond geometry (Å, º)

**Table:**

*D*—H···*A*	*D*—H	H···*A*	*D*···*A*	*D*—H···*A*
N2—H21···O5	0.89 (2)	1.93 (2)	2.819 (2)	177 (3)
N2—H22···O4^i^	0.94 (2)	1.65 (2)	2.583 (2)	177 (3)
O3—H17···O6	0.85 (2)	1.82 (2)	2.669 (2)	177 (3)
O6—H1*W*···O5^ii^	0.83 (2)	1.95 (2)	2.768 (2)	169 (3)
O6—H2*W*···O1^iii^	0.83 (2)	2.11 (2)	2.944 (2)	178 (3)

Symmetry codes: (i) −*x*+1, −*y*+2, −*z*+2; (ii) −*x*, −*y*+1, −*z*+1; (iii) −*x*, −*y*, −*z*.

### 4-(4-Nitrophenyl)piperazin-1-ium 4-methylbenzoate monohydrate (IV) Crystal data

**Table:**

C_10_H_14_N_3_O_2_^+^·C_8_H_7_O_2_^−^·H_2_O	*Z* = 2
*M**_r_* = 361.39	*F*(000) = 384
Triclinic, *P*1	*D*_x_ = 1.319 Mg m^−^^3^
Hall symbol: -P 1	Mo *K*α radiation, λ = 0.71073 Å
*a* = 6.1136 (5) Å	Cell parameters from 5980 reflections
*b* = 7.6965 (7) Å	θ = 3.1–25.4°
*c* = 19.708 (2) Å	µ = 0.10 mm^−^^1^
α = 79.577 (8)°	*T* = 293 K
β = 87.162 (8)°	Plate, yellow
γ = 86.699 (8)°	0.48 × 0.26 × 0.02 mm
*V* = 909.79 (15) Å^3^	

### 4-(4-Nitrophenyl)piperazin-1-ium 4-methylbenzoate monohydrate (IV) Data collection

**Table:**

Oxford Diffraction Xcalibur diffractometer	1911 reflections with *I* > 2σ(*I*)
ω scans	*R*_int_ = 0.019
Absorption correction: multi-scan (CrysAlis RED; Oxford Diffraction, 2009)	θ_max_ = 25.4°, θ_min_ = 3.1°
*T*_min_ = 0.970, *T*_max_ = 0.998	*h* = −5→7
5980 measured reflections	*k* = −8→9
3347 independent reflections	*l* = −23→21

### 4-(4-Nitrophenyl)piperazin-1-ium 4-methylbenzoate monohydrate (IV) Refinement

**Table:**

Refinement on *F*^2^	0 constraints
Least-squares matrix: full	Hydrogen site location: mixed
*R*[*F*^2^ > 2σ(*F*^2^)] = 0.053	H atoms treated by a mixture of independent and constrained refinement
*wR*(*F*^2^) = 0.138	*w* = 1/[σ^2^(*F*_o_^2^) + (0.0554*P*)^2^ + 0.2441*P*] where *P* = (*F*_o_^2^ + 2*F*_c_^2^)/3
*S* = 1.01	(Δ/σ)_max_ < 0.001
3343 reflections	Δρ_max_ = 0.20 e Å^−^^3^
248 parameters	Δρ_min_ = −0.16 e Å^−^^3^
4 restraints	

### 4-(4-Nitrophenyl)piperazin-1-ium 4-methylbenzoate monohydrate (IV) Special details

**Table:**

Geometry. All esds (except the esd in the dihedral angle between two l.s. planes) are estimated using the full covariance matrix. The cell esds are taken into account individually in the estimation of esds in distances, angles and torsion angles; correlations between esds in cell parameters are only used when they are defined by crystal symmetry. An approximate (isotropic) treatment of cell esds is used for estimating esds involving l.s. planes.

### 4-(4-Nitrophenyl)piperazin-1-ium 4-methylbenzoate monohydrate (IV) Fractional atomic coordinates and isotropic or equivalent isotropic displacement parameters (Å^2^)

**Table:**

	*x*	*y*	*z*	*U*_iso_*/*U*_eq_	
O1	0.7597 (5)	−0.1408 (4)	0.52918 (12)	0.1114 (9)	
O2	1.0599 (5)	−0.2808 (4)	0.50875 (13)	0.1312 (11)	
N1	0.8270 (3)	0.0591 (2)	0.20408 (9)	0.0426 (5)	
N2	0.8376 (3)	0.2441 (3)	0.06365 (10)	0.0492 (5)	
N3	0.9029 (6)	−0.1858 (4)	0.48992 (13)	0.0795 (8)	
C1	0.8503 (4)	0.0072 (3)	0.27523 (12)	0.0421 (6)	
C2	0.6848 (5)	0.0416 (4)	0.32264 (13)	0.0620 (8)	
H2	0.559385	0.107832	0.306884	0.074*	
C3	0.7020 (5)	−0.0200 (4)	0.39241 (13)	0.0660 (8)	
H3	0.58958	0.005008	0.423273	0.079*	
C4	0.8844 (5)	−0.1175 (4)	0.41582 (13)	0.0589 (7)	
C5	1.0511 (5)	−0.1516 (4)	0.37142 (15)	0.0698 (8)	
H5	1.175889	−0.217226	0.388096	0.084*	
C6	1.0361 (4)	−0.0894 (4)	0.30170 (13)	0.0606 (8)	
H6	1.152207	−0.112351	0.271761	0.073*	
C7	0.6682 (4)	0.2049 (3)	0.18122 (12)	0.0490 (6)	
H7A	0.721093	0.313511	0.191295	0.059*	
H7B	0.530333	0.182601	0.207033	0.059*	
C8	0.6287 (4)	0.2287 (4)	0.10502 (12)	0.0546 (7)	
H8A	0.553149	0.128385	0.095886	0.066*	
H8B	0.535474	0.334375	0.091454	0.066*	
C9	0.9774 (4)	0.0810 (3)	0.08466 (12)	0.0525 (7)	
H9A	1.113597	0.087916	0.057129	0.063*	
H9B	0.902753	−0.020822	0.076595	0.063*	
C10	1.0256 (4)	0.0595 (3)	0.15971 (12)	0.0499 (6)	
H10A	1.110342	−0.050791	0.173238	0.06*	
H10B	1.11409	0.155146	0.166426	0.06*	
O3	0.2656 (3)	0.7140 (3)	0.07107 (9)	0.0598 (5)	
O4	0.0053 (3)	0.5296 (3)	0.11199 (9)	0.0620 (5)	
C11	0.2728 (4)	0.5764 (3)	0.18855 (12)	0.0399 (6)	
C12	0.4646 (4)	0.6537 (3)	0.19936 (13)	0.0484 (6)	
H12	0.533917	0.726876	0.162881	0.058*	
C13	0.5535 (4)	0.6230 (3)	0.26365 (14)	0.0575 (7)	
H13	0.68258	0.675862	0.269499	0.069*	
C14	0.4567 (5)	0.5162 (3)	0.31955 (14)	0.0562 (7)	
C15	0.2645 (4)	0.4395 (4)	0.30874 (14)	0.0584 (7)	
H15	0.195058	0.366932	0.345395	0.07*	
C16	0.1748 (4)	0.4689 (3)	0.24468 (13)	0.0496 (6)	
H16	0.045866	0.41565	0.238897	0.06*	
C17	0.1749 (4)	0.6080 (3)	0.11893 (13)	0.0437 (6)	
C18	0.5550 (6)	0.4855 (4)	0.38973 (16)	0.0863 (10)	
H18A	0.442848	0.502929	0.424033	0.129*	
H18B	0.667715	0.567332	0.389742	0.129*	
H18C	0.617002	0.366684	0.400046	0.129*	
O5	0.7006 (3)	0.7136 (5)	0.02422 (13)	0.1211 (12)	
H21	0.903 (6)	0.338 (4)	0.073 (2)	0.145*	
H22	0.807 (6)	0.256 (5)	0.0183 (11)	0.145*	
H1W	0.792 (5)	0.667 (5)	0.0534 (17)	0.145*	
H2W	0.576 (4)	0.720 (6)	0.045 (2)	0.145*	

### 4-(4-Nitrophenyl)piperazin-1-ium 4-methylbenzoate monohydrate (IV) Atomic displacement parameters (Å^2^)

**Table:**

	*U*^11^	*U*^22^	*U*^33^	*U*^12^	*U*^13^	*U*^23^
O1	0.145 (2)	0.132 (2)	0.0477 (14)	0.0055 (18)	0.0100 (15)	−0.0001 (14)
O2	0.153 (2)	0.155 (3)	0.0702 (17)	0.044 (2)	−0.0390 (17)	0.0148 (17)
N1	0.0427 (11)	0.0478 (12)	0.0358 (11)	0.0059 (9)	−0.0018 (9)	−0.0064 (9)
N2	0.0540 (13)	0.0562 (14)	0.0357 (11)	0.0002 (11)	−0.0042 (10)	−0.0041 (10)
N3	0.111 (2)	0.078 (2)	0.0469 (17)	−0.0053 (17)	−0.0113 (16)	−0.0005 (14)
C1	0.0489 (14)	0.0408 (14)	0.0377 (14)	0.0000 (11)	−0.0047 (11)	−0.0100 (11)
C2	0.0675 (17)	0.0685 (19)	0.0442 (16)	0.0202 (15)	0.0004 (13)	−0.0028 (14)
C3	0.083 (2)	0.072 (2)	0.0394 (16)	0.0130 (17)	0.0050 (14)	−0.0057 (14)
C4	0.085 (2)	0.0556 (17)	0.0352 (15)	0.0002 (16)	−0.0113 (14)	−0.0044 (13)
C5	0.0719 (19)	0.083 (2)	0.0515 (19)	0.0176 (17)	−0.0203 (15)	−0.0060 (16)
C6	0.0564 (16)	0.078 (2)	0.0448 (16)	0.0167 (15)	−0.0066 (13)	−0.0092 (14)
C7	0.0428 (14)	0.0616 (17)	0.0401 (14)	0.0080 (12)	−0.0034 (11)	−0.0050 (12)
C8	0.0441 (14)	0.0706 (18)	0.0468 (16)	0.0043 (13)	−0.0071 (12)	−0.0052 (13)
C9	0.0597 (16)	0.0579 (17)	0.0385 (15)	0.0061 (13)	0.0028 (12)	−0.0086 (12)
C10	0.0499 (15)	0.0558 (16)	0.0417 (15)	0.0138 (12)	−0.0008 (12)	−0.0080 (12)
O3	0.0574 (11)	0.0779 (13)	0.0404 (10)	−0.0028 (10)	−0.0074 (8)	0.0006 (9)
O4	0.0577 (11)	0.0758 (13)	0.0557 (12)	−0.0102 (10)	−0.0167 (9)	−0.0143 (10)
C11	0.0404 (13)	0.0387 (13)	0.0413 (14)	0.0068 (11)	−0.0074 (11)	−0.0103 (11)
C12	0.0513 (15)	0.0449 (15)	0.0490 (16)	−0.0006 (12)	−0.0065 (12)	−0.0072 (12)
C13	0.0547 (16)	0.0533 (17)	0.0679 (19)	0.0004 (13)	−0.0224 (14)	−0.0158 (15)
C14	0.0707 (18)	0.0497 (16)	0.0498 (17)	0.0066 (14)	−0.0233 (14)	−0.0103 (13)
C15	0.0706 (18)	0.0570 (17)	0.0453 (16)	−0.0042 (14)	−0.0099 (13)	−0.0007 (13)
C16	0.0488 (15)	0.0518 (16)	0.0485 (16)	−0.0039 (13)	−0.0089 (12)	−0.0074 (13)
C17	0.0427 (14)	0.0473 (15)	0.0420 (15)	0.0071 (12)	−0.0042 (12)	−0.0121 (12)
C18	0.114 (3)	0.079 (2)	0.068 (2)	−0.003 (2)	−0.0467 (19)	−0.0066 (17)
O5	0.0602 (14)	0.210 (3)	0.0697 (16)	−0.0101 (18)	−0.0093 (12)	0.0407 (18)

### 4-(4-Nitrophenyl)piperazin-1-ium 4-methylbenzoate monohydrate (IV) Geometric parameters (Å, º)

**Table:**

O1—N3	1.216 (3)	C9—C10	1.500 (3)
O2—N3	1.204 (3)	C9—H9A	0.97
N1—C1	1.399 (3)	C9—H9B	0.97
N1—C10	1.460 (3)	C10—H10A	0.97
N1—C7	1.463 (3)	C10—H10B	0.97
N2—C8	1.480 (3)	O3—O3	0.000 (5)
N2—C9	1.483 (3)	O3—C17	1.259 (3)
N2—H21	0.892 (19)	O4—C17	1.254 (3)
N2—H22	0.908 (19)	C11—C12	1.387 (3)
N3—C4	1.468 (3)	C11—C16	1.389 (3)
C1—C2	1.390 (3)	C11—C17	1.498 (3)
C1—C6	1.391 (3)	C12—C13	1.379 (3)
C2—C3	1.378 (4)	C12—H12	0.93
C2—H2	0.93	C13—C14	1.380 (4)
C3—C4	1.360 (4)	C13—H13	0.93
C3—H3	0.93	C14—C15	1.387 (4)
C4—C5	1.356 (4)	C14—C18	1.509 (4)
C5—C6	1.377 (4)	C15—C16	1.377 (3)
C5—H5	0.93	C15—H15	0.93
C6—H6	0.93	C16—H16	0.93
C7—C8	1.509 (3)	C18—H18A	0.96
C7—H7A	0.97	C18—H18B	0.96
C7—H7B	0.97	C18—H18C	0.96
C8—H8A	0.97	O5—H1W	0.843 (19)
C8—H8B	0.97	O5—H2W	0.854 (19)
			
C1—N1—C10	117.39 (18)	N2—C9—H9A	109.6
C1—N1—C7	117.37 (18)	C10—C9—H9A	109.6
C10—N1—C7	113.94 (18)	N2—C9—H9B	109.6
C8—N2—C9	108.7 (2)	C10—C9—H9B	109.6
C8—N2—H21	107 (3)	H9A—C9—H9B	108.2
C9—N2—H21	110 (3)	N1—C10—C9	112.7 (2)
C8—N2—H22	109 (3)	N1—C10—H10A	109.1
C9—N2—H22	110 (3)	C9—C10—H10A	109.1
H21—N2—H22	112 (4)	N1—C10—H10B	109.1
O2—N3—O1	123.5 (3)	C9—C10—H10B	109.1
O2—N3—C4	118.5 (3)	H10A—C10—H10B	107.8
O1—N3—C4	117.9 (3)	O3—O3—C17	0 (10)
C2—C1—C6	116.8 (2)	C12—C11—C16	117.5 (2)
C2—C1—N1	121.7 (2)	C12—C11—C17	121.2 (2)
C6—C1—N1	121.4 (2)	C16—C11—C17	121.2 (2)
C3—C2—C1	121.6 (3)	C13—C12—C11	120.7 (2)
C3—C2—H2	119.2	C13—C12—H12	119.7
C1—C2—H2	119.2	C11—C12—H12	119.7
C4—C3—C2	119.6 (3)	C12—C13—C14	122.0 (2)
C4—C3—H3	120.2	C12—C13—H13	119
C2—C3—H3	120.2	C14—C13—H13	119
C5—C4—C3	120.7 (2)	C13—C14—C15	117.3 (2)
C5—C4—N3	119.3 (3)	C13—C14—C18	121.3 (3)
C3—C4—N3	120.0 (3)	C15—C14—C18	121.4 (3)
C4—C5—C6	120.1 (3)	C16—C15—C14	121.2 (3)
C4—C5—H5	119.9	C16—C15—H15	119.4
C6—C5—H5	119.9	C14—C15—H15	119.4
C5—C6—C1	121.2 (2)	C15—C16—C11	121.3 (2)
C5—C6—H6	119.4	C15—C16—H16	119.3
C1—C6—H6	119.4	C11—C16—H16	119.3
N1—C7—C8	112.6 (2)	O4—C17—O3	123.8 (2)
N1—C7—H7A	109.1	O4—C17—O3	123.8 (2)
C8—C7—H7A	109.1	O3—C17—O3	0.0 (2)
N1—C7—H7B	109.1	O4—C17—C11	118.1 (2)
C8—C7—H7B	109.1	O3—C17—C11	118.0 (2)
H7A—C7—H7B	107.8	O3—C17—C11	118.0 (2)
N2—C8—C7	111.1 (2)	C14—C18—H18A	109.5
N2—C8—H8A	109.4	C14—C18—H18B	109.5
C7—C8—H8A	109.4	H18A—C18—H18B	109.5
N2—C8—H8B	109.4	C14—C18—H18C	109.5
C7—C8—H8B	109.4	H18A—C18—H18C	109.5
H8A—C8—H8B	108	H18B—C18—H18C	109.5
N2—C9—C10	110.1 (2)	H1W—O5—H2W	108 (4)
			
C10—N1—C1—C2	164.4 (2)	C8—N2—C9—C10	−60.7 (3)
C7—N1—C1—C2	22.9 (3)	C1—N1—C10—C9	168.3 (2)
C10—N1—C1—C6	−18.9 (3)	C7—N1—C10—C9	−48.9 (3)
C7—N1—C1—C6	−160.4 (2)	N2—C9—C10—N1	55.7 (3)
C6—C1—C2—C3	−1.2 (4)	C16—C11—C12—C13	0.3 (3)
N1—C1—C2—C3	175.6 (3)	C17—C11—C12—C13	−179.7 (2)
C1—C2—C3—C4	−0.3 (5)	C11—C12—C13—C14	−0.3 (4)
C2—C3—C4—C5	1.3 (5)	C12—C13—C14—C15	0.1 (4)
C2—C3—C4—N3	−179.1 (3)	C12—C13—C14—C18	−179.4 (3)
O2—N3—C4—C5	−5.0 (4)	C13—C14—C15—C16	0.1 (4)
O1—N3—C4—C5	173.8 (3)	C18—C14—C15—C16	179.6 (3)
O2—N3—C4—C3	175.5 (3)	C14—C15—C16—C11	−0.1 (4)
O1—N3—C4—C3	−5.7 (4)	C12—C11—C16—C15	−0.1 (4)
C3—C4—C5—C6	−0.8 (5)	C17—C11—C16—C15	179.9 (2)
N3—C4—C5—C6	179.6 (3)	O3—O3—C17—O4	0.00 (14)
C4—C5—C6—C1	−0.8 (5)	O3—O3—C17—C11	0.0 (2)
C2—C1—C6—C5	1.8 (4)	C12—C11—C17—O4	177.7 (2)
N1—C1—C6—C5	−175.0 (3)	C16—C11—C17—O4	−2.4 (3)
C1—N1—C7—C8	−170.1 (2)	C12—C11—C17—O3	−3.5 (3)
C10—N1—C7—C8	47.0 (3)	C16—C11—C17—O3	176.4 (2)
C9—N2—C8—C7	59.5 (3)	C12—C11—C17—O3	−3.5 (3)
N1—C7—C8—N2	−52.7 (3)	C16—C11—C17—O3	176.4 (2)

### 4-(4-Nitrophenyl)piperazin-1-ium 4-methylbenzoate monohydrate (IV) Hydrogen-bond geometry (Å, º)

*Cg*3 is the centroids of the C11–C16 ring.

**Table:**

*D*—H···*A*	*D*—H	H···*A*	*D*···*A*	*D*—H···*A*
N2—H21···O4^i^	0.89 (2)	1.93 (2)	2.811 (3)	167 (4)
N2—H22···O3^ii^	0.91 (2)	1.81 (2)	2.717 (3)	177 (4)
C3—H3···O1^iii^	0.93	2.54	3.427 (4)	161
C9—H9*A*···O5^iv^	0.97	2.31	3.113 (3)	140
O5—H1*W*···O4^i^	0.84 (2)	1.92 (2)	2.756 (3)	171 (4)
O5—H2*W*···O3	0.85 (2)	1.94 (2)	2.772 (3)	164 (4)
C6—H6···*Cg*3^v^	0.93	2.93	3.590 (3)	129

Symmetry codes: (i) *x*+1, *y*, *z*; (ii) −*x*+1, −*y*+1, −*z*; (iii) −*x*+1, −*y*, −*z*+1; (iv) −*x*+2, −*y*+1, −*z*; (v) *x*+1, *y*−1, *z*.

### 4-(4-Nitrophenyl)piperazin-1-ium 4-methoxybenzoate hemihydrate (V) Crystal data

**Table:**

2C_10_H_14_N_3_O_2_^+^·2C_8_H_7_O_3_^−^·H_2_O	*F*(000) = 1560
*M**_r_* = 736.77	*D*_x_ = 1.328 Mg m^−^^3^
Monoclinic, *P*2_1_/*c*	Mo *K*α radiation, λ = 0.71073 Å
Hall symbol: -P 2ybc	Cell parameters from 2899 reflections
*a* = 15.808 (1) Å	θ = 2.6–25.3°
*b* = 7.5198 (7) Å	µ = 0.1 mm^−^^1^
*c* = 31.020 (2) Å	*T* = 293 K
β = 92.561 (7)°	Prism, orange
*V* = 3683.8 (5) Å^3^	0.5 × 0.36 × 0.36 mm
*Z* = 4	

### 4-(4-Nitrophenyl)piperazin-1-ium 4-methoxybenzoate hemihydrate (V) Data collection

**Table:**

Oxford Diffraction Xcalibur diffractometer	2602 reflections with *I* > 2σ(*I*)
ω scans	*R*_int_ = 0.066
Absorption correction: multi-scan (CrysAlis RED; Oxford Diffraction, 2009)	θ_max_ = 25.3°, θ_min_ = 2.6°
*T*_min_ = 0.958, *T*_max_ = 0.965	*h* = −19→18
15326 measured reflections	*k* = −9→8
6718 independent reflections	*l* = −37→33

### 4-(4-Nitrophenyl)piperazin-1-ium 4-methoxybenzoate hemihydrate (V) Refinement

**Table:**

Refinement on *F*^2^	Hydrogen site location: mixed
Least-squares matrix: full	H atoms treated by a mixture of independent and constrained refinement
*R*[*F*^2^ > 2σ(*F*^2^)] = 0.074	*w* = 1/[σ^2^(*F*_o_^2^) + (0.0554*P*)^2^ + 0.9198*P*] where *P* = (*F*_o_^2^ + 2*F*_c_^2^)/3
*wR*(*F*^2^) = 0.169	(Δ/σ)_max_ < 0.001
*S* = 1.00	Δρ_max_ = 0.27 e Å^−^^3^
6715 reflections	Δρ_min_ = −0.18 e Å^−^^3^
507 parameters	Extinction correction: SHELXL2018/3 (Sheldrick 2015b), Fc^*^=kFc[1+0.001xFc^2^λ^3^/sin(2θ)]^-1/4^
45 restraints	Extinction coefficient: 0.0029 (4)
0 constraints	

### 4-(4-Nitrophenyl)piperazin-1-ium 4-methoxybenzoate hemihydrate (V) Special details

**Table:**

Geometry. All esds (except the esd in the dihedral angle between two l.s. planes) are estimated using the full covariance matrix. The cell esds are taken into account individually in the estimation of esds in distances, angles and torsion angles; correlations between esds in cell parameters are only used when they are defined by crystal symmetry. An approximate (isotropic) treatment of cell esds is used for estimating esds involving l.s. planes.

### 4-(4-Nitrophenyl)piperazin-1-ium 4-methoxybenzoate hemihydrate (V) Fractional atomic coordinates and isotropic or equivalent isotropic displacement parameters (Å^2^)

**Table:**

	*x*	*y*	*z*	*U*_iso_*/*U*_eq_	Occ. (<1)
C1	0.2115 (2)	0.2769 (5)	0.15386 (13)	0.0456 (11)	
C2	0.2042 (3)	0.2439 (6)	0.19789 (14)	0.0687 (14)	
H2	0.151236	0.21805	0.208209	0.082*	
C3	0.2734 (3)	0.2487 (7)	0.22634 (14)	0.0860 (16)	
H3	0.26712	0.22698	0.255532	0.103*	
C4	0.3512 (3)	0.2855 (7)	0.21153 (15)	0.0702 (14)	
C5	0.3613 (3)	0.3157 (6)	0.16906 (15)	0.0722 (14)	
H5	0.414916	0.339042	0.159279	0.087*	
C6	0.2925 (3)	0.3119 (6)	0.14031 (13)	0.0598 (12)	
H6	0.300335	0.333225	0.111231	0.072*	
C7	0.1555 (2)	0.2450 (6)	0.07998 (12)	0.0599 (12)	
H7A	0.166523	0.119002	0.076636	0.072*	
H7B	0.205192	0.309359	0.07138	0.072*	
C8	0.0813 (3)	0.2965 (6)	0.05086 (13)	0.0690 (13)	
H8A	0.075136	0.42483	0.050937	0.083*	
H8B	0.09152	0.259489	0.021595	0.083*	
C9	−0.0117 (3)	0.2610 (6)	0.10976 (14)	0.0669 (13)	
H9A	−0.062504	0.202151	0.118906	0.08*	
H9B	−0.020149	0.38837	0.112112	0.08*	
C10	0.0624 (2)	0.2059 (6)	0.13852 (12)	0.0631 (13)	
H10A	0.052793	0.242915	0.16785	0.076*	
H10B	0.066876	0.077208	0.138331	0.076*	
C11	0.9721 (3)	0.2971 (6)	0.30361 (13)	0.0510 (11)	
C12	0.8980 (3)	0.2023 (6)	0.30443 (12)	0.0563 (12)	
H12	0.888658	0.13067	0.328178	0.068*	
C13	0.8365 (3)	0.2095 (6)	0.27110 (14)	0.0632 (13)	
H13	0.78756	0.14142	0.272281	0.076*	
C14	0.8487 (3)	0.3182 (6)	0.23643 (14)	0.0628 (12)	
C15	0.9229 (3)	0.4129 (6)	0.23467 (13)	0.0649 (13)	
H15	0.93208	0.484494	0.210883	0.078*	
C16	0.9839 (3)	0.4028 (6)	0.26775 (14)	0.0626 (13)	
H16	1.033592	0.46798	0.26601	0.075*	
C17	1.0355 (3)	0.2887 (7)	0.34129 (16)	0.0608 (13)	
C18	0.7146 (3)	0.2457 (8)	0.20218 (17)	0.118 (2)	
H18C	0.681472	0.273815	0.176387	0.178*	
H18B	0.725759	0.120242	0.20308	0.178*	
H18A	0.684002	0.279268	0.226948	0.178*	
C19	0.3816 (3)	0.5044 (6)	−0.09170 (13)	0.0503 (11)	
C20	0.3778 (3)	0.6432 (6)	−0.06180 (13)	0.0582 (12)	
H20	0.326082	0.697741	−0.057457	0.07*	
C21	0.4478 (3)	0.6998 (6)	−0.03904 (13)	0.0603 (12)	
H21	0.443054	0.790435	−0.018877	0.072*	
C22	0.5251 (2)	0.6260 (6)	−0.04523 (13)	0.0499 (11)	
C23	0.5330 (3)	0.4942 (6)	−0.07542 (14)	0.0610 (12)	
H23	0.58581	0.445412	−0.080183	0.073*	
C24	0.4629 (3)	0.4354 (6)	−0.09836 (13)	0.0623 (13)	
H24	0.468943	0.347143	−0.119	0.075*	
C25	0.3099 (3)	0.2720 (7)	−0.13618 (14)	0.0722 (14)	
H25A	0.36547	0.248859	−0.147048	0.087*	
H25B	0.269401	0.27516	−0.160582	0.087*	
C26	0.2867 (3)	0.1245 (6)	−0.10589 (14)	0.0694 (13)	
H26A	0.284623	0.011999	−0.121195	0.083*	
H26B	0.329107	0.115475	−0.082393	0.083*	
C27	0.1998 (2)	0.3414 (6)	−0.06853 (12)	0.0579 (12)	
H27A	0.237409	0.345068	−0.042968	0.07*	
H27B	0.142682	0.365292	−0.059877	0.07*	
C28	0.2258 (2)	0.4799 (6)	−0.10009 (13)	0.0583 (12)	
H28A	0.186099	0.480675	−0.124849	0.07*	
H28B	0.224623	0.596261	−0.086644	0.07*	
C29	0.2315 (2)	0.8165 (5)	0.03520 (12)	0.0442 (10)	
C30	0.3063 (2)	0.9094 (5)	0.03221 (13)	0.0517 (11)	
H30	0.319956	0.957242	0.005761	0.062*	
C31	0.3609 (2)	0.9330 (6)	0.06743 (15)	0.0597 (12)	
H31	0.411473	0.994162	0.064463	0.072*	
C32	0.3412 (3)	0.8662 (6)	0.10717 (15)	0.0570 (12)	
C33	0.2661 (3)	0.7752 (6)	0.11108 (13)	0.0590 (12)	
H33	0.251489	0.731519	0.137782	0.071*	
C34	0.2130 (2)	0.7495 (5)	0.07521 (13)	0.0524 (11)	
H34	0.163238	0.685277	0.077981	0.063*	
C35	0.1734 (3)	0.7879 (6)	−0.00372 (14)	0.0459 (11)	
C36	0.3801 (3)	0.8360 (7)	0.18157 (17)	0.1098 (19)	
H36C	0.42611	0.863024	0.201777	0.165*	
H36B	0.329805	0.895356	0.190161	0.165*	
H36A	0.370656	0.709953	0.181018	0.165*	
N1	0.14171 (19)	0.2823 (4)	0.12508 (10)	0.0475 (9)	
N2	0.0026 (2)	0.2144 (5)	0.06465 (13)	0.0581 (10)	
N3	0.4265 (6)	0.2464 (13)	0.2410 (3)	0.075 (2)	0.519 (6)
N3'	0.4213 (6)	0.3345 (15)	0.2420 (3)	0.075 (2)	0.481 (6)
N4	0.3106 (2)	0.4437 (5)	−0.11425 (10)	0.0583 (10)	
N5	0.2034 (2)	0.1639 (5)	−0.08869 (12)	0.0612 (10)	
N6	0.5983 (2)	0.6796 (6)	−0.01867 (13)	0.0650 (11)	
O1	0.4960 (7)	0.2571 (14)	0.2266 (4)	0.099 (2)	0.519 (6)
O1'	0.4904 (8)	0.3633 (14)	0.2283 (4)	0.099 (2)	0.481 (6)
O2	0.4177 (6)	0.2116 (13)	0.2788 (3)	0.097 (2)	0.519 (6)
O2'	0.4086 (6)	0.3408 (15)	0.2797 (3)	0.097 (2)	0.481 (6)
O3	1.1003 (2)	0.3818 (4)	0.33942 (9)	0.0826 (10)	
O4	1.0196 (2)	0.1889 (4)	0.37194 (10)	0.0789 (10)	
O5	0.7922 (2)	0.3398 (5)	0.20234 (9)	0.0895 (11)	
O6	0.58811 (19)	0.7838 (5)	0.01114 (11)	0.0827 (11)	
O7	0.66853 (19)	0.6203 (5)	−0.02668 (10)	0.0881 (11)	
O8	0.10807 (17)	0.6951 (4)	0.00037 (8)	0.0590 (8)	
O9	0.19207 (16)	0.8596 (4)	−0.03853 (9)	0.0593 (8)	
O10	0.40056 (18)	0.8950 (4)	0.13979 (10)	0.0790 (10)	
O11	1.00751 (19)	0.3517 (4)	0.44766 (10)	0.0649 (9)	
H21N	−0.0388 (19)	0.252 (5)	0.0468 (11)	0.078*	
H22N	0.007 (3)	0.099 (3)	0.0609 (12)	0.078*	
H51N	0.190 (2)	0.077 (4)	−0.0717 (11)	0.078*	
H52N	0.164 (2)	0.153 (6)	−0.1110 (9)	0.078*	
H11O	1.010 (3)	0.296 (5)	0.4244 (9)	0.078*	
H12O	0.969 (2)	0.305 (5)	0.4618 (12)	0.078*	

### 4-(4-Nitrophenyl)piperazin-1-ium 4-methoxybenzoate hemihydrate (V) Atomic displacement parameters (Å^2^)

**Table:**

	*U*^11^	*U*^22^	*U*^33^	*U*^12^	*U*^13^	*U*^23^
C1	0.043 (3)	0.040 (3)	0.054 (3)	0.002 (2)	0.000 (2)	−0.002 (2)
C2	0.056 (3)	0.100 (4)	0.051 (3)	−0.008 (3)	0.007 (3)	0.001 (3)
C3	0.073 (3)	0.145 (5)	0.039 (3)	−0.011 (4)	−0.005 (3)	0.004 (3)
C4	0.052 (3)	0.108 (4)	0.050 (3)	−0.009 (3)	−0.013 (2)	0.001 (3)
C5	0.052 (3)	0.107 (4)	0.057 (3)	−0.017 (3)	−0.001 (3)	0.005 (3)
C6	0.049 (3)	0.082 (4)	0.048 (3)	−0.005 (3)	−0.006 (2)	0.009 (2)
C7	0.050 (3)	0.082 (3)	0.047 (3)	−0.009 (3)	−0.002 (2)	0.002 (2)
C8	0.064 (3)	0.077 (3)	0.064 (3)	−0.023 (3)	−0.018 (2)	0.017 (3)
C9	0.052 (3)	0.076 (4)	0.073 (4)	−0.003 (3)	−0.003 (2)	−0.015 (3)
C10	0.052 (3)	0.089 (4)	0.048 (3)	−0.013 (3)	0.000 (2)	−0.003 (2)
C11	0.055 (3)	0.049 (3)	0.049 (3)	0.004 (3)	0.004 (2)	0.002 (2)
C12	0.071 (3)	0.059 (3)	0.039 (3)	0.007 (3)	0.006 (2)	0.008 (2)
C13	0.058 (3)	0.074 (4)	0.058 (3)	−0.010 (3)	0.000 (2)	0.010 (3)
C14	0.064 (3)	0.071 (4)	0.051 (3)	0.007 (3)	−0.012 (3)	0.004 (3)
C15	0.068 (3)	0.070 (4)	0.056 (3)	−0.009 (3)	−0.004 (3)	0.018 (2)
C16	0.058 (3)	0.064 (3)	0.065 (3)	−0.009 (3)	0.000 (3)	0.009 (3)
C17	0.063 (3)	0.057 (4)	0.062 (3)	0.014 (3)	−0.010 (3)	−0.011 (3)
C18	0.103 (5)	0.148 (6)	0.100 (4)	−0.040 (4)	−0.047 (3)	0.023 (4)
C19	0.044 (3)	0.065 (3)	0.042 (3)	−0.002 (3)	0.007 (2)	0.009 (2)
C20	0.038 (3)	0.071 (3)	0.066 (3)	0.003 (3)	0.002 (2)	−0.004 (3)
C21	0.046 (3)	0.071 (3)	0.064 (3)	−0.002 (3)	0.008 (2)	−0.009 (2)
C22	0.035 (3)	0.064 (3)	0.051 (3)	−0.007 (2)	0.002 (2)	0.009 (2)
C23	0.038 (3)	0.068 (3)	0.077 (3)	0.004 (3)	0.012 (2)	0.005 (3)
C24	0.050 (3)	0.067 (3)	0.071 (3)	−0.003 (3)	0.015 (3)	−0.011 (2)
C25	0.063 (3)	0.100 (4)	0.054 (3)	−0.009 (3)	0.006 (2)	−0.023 (3)
C26	0.069 (3)	0.068 (4)	0.070 (3)	0.003 (3)	−0.008 (3)	−0.015 (3)
C27	0.049 (3)	0.070 (3)	0.054 (3)	−0.004 (3)	−0.003 (2)	−0.012 (3)
C28	0.042 (3)	0.063 (3)	0.069 (3)	−0.006 (2)	−0.008 (2)	0.001 (3)
C29	0.043 (2)	0.045 (3)	0.045 (3)	0.005 (2)	0.004 (2)	−0.003 (2)
C30	0.044 (3)	0.063 (3)	0.049 (3)	0.006 (2)	0.004 (2)	0.007 (2)
C31	0.044 (3)	0.071 (3)	0.064 (3)	−0.005 (2)	−0.005 (2)	−0.001 (3)
C32	0.053 (3)	0.064 (3)	0.053 (3)	0.012 (3)	−0.013 (3)	−0.008 (3)
C33	0.062 (3)	0.067 (3)	0.048 (3)	−0.001 (3)	−0.005 (2)	0.005 (2)
C34	0.052 (3)	0.055 (3)	0.050 (3)	−0.004 (2)	−0.002 (2)	0.006 (2)
C35	0.048 (3)	0.040 (3)	0.050 (3)	0.009 (2)	−0.005 (2)	−0.002 (2)
C36	0.117 (5)	0.125 (5)	0.083 (4)	0.008 (4)	−0.035 (3)	−0.004 (4)
N1	0.041 (2)	0.055 (2)	0.046 (2)	−0.0042 (18)	−0.0010 (17)	−0.0018 (17)
N2	0.055 (3)	0.047 (2)	0.070 (3)	−0.005 (2)	−0.0165 (19)	0.004 (2)
N3	0.072 (3)	0.081 (4)	0.070 (3)	−0.002 (3)	−0.004 (2)	−0.001 (3)
N3'	0.072 (3)	0.081 (4)	0.070 (3)	−0.002 (3)	−0.004 (2)	−0.001 (3)
N4	0.051 (2)	0.072 (3)	0.052 (2)	−0.005 (2)	0.0048 (19)	−0.002 (2)
N5	0.059 (3)	0.063 (3)	0.061 (3)	−0.012 (2)	−0.009 (2)	0.010 (2)
N6	0.048 (3)	0.084 (3)	0.064 (3)	−0.012 (3)	0.003 (2)	0.021 (2)
O1	0.075 (3)	0.127 (7)	0.092 (3)	−0.011 (5)	−0.020 (2)	−0.001 (6)
O1'	0.075 (3)	0.127 (7)	0.092 (3)	−0.011 (5)	−0.020 (2)	−0.001 (6)
O2	0.097 (3)	0.131 (6)	0.062 (3)	−0.003 (5)	−0.015 (2)	0.001 (5)
O2'	0.097 (3)	0.131 (6)	0.062 (3)	−0.003 (5)	−0.015 (2)	0.001 (5)
O3	0.073 (2)	0.088 (3)	0.084 (2)	−0.010 (2)	−0.0291 (18)	0.0096 (18)
O4	0.106 (3)	0.069 (2)	0.060 (2)	0.003 (2)	−0.0170 (18)	0.0134 (18)
O5	0.082 (2)	0.115 (3)	0.070 (2)	−0.013 (2)	−0.0256 (19)	0.0253 (19)
O6	0.072 (2)	0.110 (3)	0.066 (2)	−0.023 (2)	0.0016 (18)	−0.002 (2)
O7	0.046 (2)	0.128 (3)	0.090 (2)	−0.001 (2)	0.0000 (18)	0.023 (2)
O8	0.0548 (18)	0.061 (2)	0.060 (2)	−0.0126 (17)	−0.0085 (14)	0.0027 (15)
O9	0.0639 (19)	0.066 (2)	0.0473 (19)	0.0027 (16)	−0.0024 (15)	0.0080 (15)
O10	0.072 (2)	0.102 (3)	0.061 (2)	0.0032 (19)	−0.0220 (18)	−0.0004 (19)
O11	0.063 (2)	0.064 (2)	0.069 (2)	0.0012 (18)	0.0089 (17)	−0.0012 (17)

### 4-(4-Nitrophenyl)piperazin-1-ium 4-methoxybenzoate hemihydrate (V) Geometric parameters (Å, º)

**Table:**

C1—N1	1.388 (4)	C21—H21	0.93
C1—C6	1.391 (5)	C22—C23	1.373 (5)
C1—C2	1.398 (5)	C22—N6	1.448 (5)
C2—C3	1.375 (6)	C23—C24	1.363 (5)
C2—H2	0.93	C23—H23	0.93
C3—C4	1.359 (6)	C24—H24	0.93
C3—H3	0.93	C25—N4	1.459 (5)
C4—C5	1.354 (5)	C25—C26	1.509 (6)
C4—N3'	1.471 (10)	C25—H25A	0.97
C4—N3	1.499 (9)	C25—H25B	0.97
C5—C6	1.375 (5)	C26—N5	1.474 (5)
C5—H5	0.93	C26—H26A	0.97
C6—H6	0.93	C26—H26B	0.97
C7—N1	1.453 (4)	C27—N5	1.476 (5)
C7—C8	1.499 (5)	C27—C28	1.499 (5)
C7—H7A	0.97	C27—H27A	0.97
C7—H7B	0.97	C27—H27B	0.97
C8—N2	1.469 (5)	C28—N4	1.455 (4)
C8—H8A	0.97	C28—H28A	0.97
C8—H8B	0.97	C28—H28B	0.97
C9—N2	1.470 (5)	C29—C30	1.379 (5)
C9—C10	1.499 (5)	C29—C34	1.383 (5)
C9—H9A	0.97	C29—C35	1.499 (5)
C9—H9B	0.97	C30—C31	1.374 (5)
C10—N1	1.457 (4)	C30—H30	0.93
C10—H10A	0.97	C31—C32	1.379 (5)
C10—H10B	0.97	C31—H31	0.93
C11—C12	1.372 (5)	C32—O10	1.366 (4)
C11—C16	1.387 (5)	C32—C33	1.380 (5)
C11—C17	1.506 (6)	C33—C34	1.377 (5)
C12—C13	1.388 (5)	C33—H33	0.93
C12—H12	0.93	C34—H34	0.93
C13—C14	1.371 (5)	C35—O9	1.254 (4)
C13—H13	0.93	C35—O8	1.258 (4)
C14—O5	1.363 (5)	C36—O10	1.421 (5)
C14—C15	1.375 (5)	C36—H36C	0.96
C15—C16	1.378 (5)	C36—H36B	0.96
C15—H15	0.93	C36—H36A	0.96
C16—H16	0.93	N2—H21N	0.884 (18)
C17—O3	1.245 (5)	N2—H22N	0.882 (18)
C17—O4	1.246 (5)	N3—O1	1.208 (9)
C18—O5	1.416 (5)	N3—O2	1.213 (9)
C18—H18C	0.96	N3'—O2'	1.196 (9)
C18—H18B	0.96	N3'—O1'	1.210 (10)
C18—H18A	0.96	N5—H51N	0.872 (18)
C19—N4	1.374 (4)	N5—H52N	0.910 (18)
C19—C20	1.399 (5)	N6—O6	1.228 (4)
C19—C24	1.410 (5)	N6—O7	1.232 (4)
C20—C21	1.354 (5)	O11—H11O	0.836 (18)
C20—H20	0.93	O11—H12O	0.838 (18)
C21—C22	1.363 (5)		
			
N1—C1—C6	121.1 (4)	C22—C23—H23	120.2
N1—C1—C2	122.4 (4)	C23—C24—C19	121.9 (4)
C6—C1—C2	116.5 (4)	C23—C24—H24	119
C3—C2—C1	121.6 (4)	C19—C24—H24	119
C3—C2—H2	119.2	N4—C25—C26	110.9 (3)
C1—C2—H2	119.2	N4—C25—H25A	109.5
C4—C3—C2	119.6 (4)	C26—C25—H25A	109.5
C4—C3—H3	120.2	N4—C25—H25B	109.5
C2—C3—H3	120.2	C26—C25—H25B	109.5
C5—C4—C3	120.7 (4)	H25A—C25—H25B	108
C5—C4—N3'	117.8 (6)	N5—C26—C25	108.9 (4)
C3—C4—N3'	120.0 (6)	N5—C26—H26A	109.9
C5—C4—N3	120.2 (6)	C25—C26—H26A	109.9
C3—C4—N3	117.3 (6)	N5—C26—H26B	109.9
C4—C5—C6	120.2 (4)	C25—C26—H26B	109.9
C4—C5—H5	119.9	H26A—C26—H26B	108.3
C6—C5—H5	119.9	N5—C27—C28	109.5 (3)
C5—C6—C1	121.3 (4)	N5—C27—H27A	109.8
C5—C6—H6	119.3	C28—C27—H27A	109.8
C1—C6—H6	119.3	N5—C27—H27B	109.8
N1—C7—C8	112.5 (3)	C28—C27—H27B	109.8
N1—C7—H7A	109.1	H27A—C27—H27B	108.2
C8—C7—H7A	109.1	N4—C28—C27	110.5 (3)
N1—C7—H7B	109.1	N4—C28—H28A	109.5
C8—C7—H7B	109.1	C27—C28—H28A	109.5
H7A—C7—H7B	107.8	N4—C28—H28B	109.5
N2—C8—C7	111.5 (3)	C27—C28—H28B	109.5
N2—C8—H8A	109.3	H28A—C28—H28B	108.1
C7—C8—H8A	109.3	C30—C29—C34	117.5 (4)
N2—C8—H8B	109.3	C30—C29—C35	120.9 (4)
C7—C8—H8B	109.3	C34—C29—C35	121.6 (4)
H8A—C8—H8B	108	C31—C30—C29	121.5 (4)
N2—C9—C10	110.5 (3)	C31—C30—H30	119.3
N2—C9—H9A	109.5	C29—C30—H30	119.3
C10—C9—H9A	109.5	C30—C31—C32	120.3 (4)
N2—C9—H9B	109.5	C30—C31—H31	119.8
C10—C9—H9B	109.5	C32—C31—H31	119.8
H9A—C9—H9B	108.1	O10—C32—C31	115.4 (4)
N1—C10—C9	112.3 (3)	O10—C32—C33	125.3 (4)
N1—C10—H10A	109.1	C31—C32—C33	119.2 (4)
C9—C10—H10A	109.1	C34—C33—C32	119.6 (4)
N1—C10—H10B	109.1	C34—C33—H33	120.2
C9—C10—H10B	109.1	C32—C33—H33	120.2
H10A—C10—H10B	107.9	C33—C34—C29	121.8 (4)
C12—C11—C16	117.2 (4)	C33—C34—H34	119.1
C12—C11—C17	120.2 (4)	C29—C34—H34	119.1
C16—C11—C17	122.5 (4)	O9—C35—O8	123.4 (4)
C11—C12—C13	122.4 (4)	O9—C35—C29	118.2 (4)
C11—C12—H12	118.8	O8—C35—C29	118.3 (4)
C13—C12—H12	118.8	O10—C36—H36C	109.5
C14—C13—C12	119.3 (4)	O10—C36—H36B	109.5
C14—C13—H13	120.4	H36C—C36—H36B	109.5
C12—C13—H13	120.4	O10—C36—H36A	109.5
O5—C14—C13	124.7 (4)	H36C—C36—H36A	109.5
O5—C14—C15	116.0 (4)	H36B—C36—H36A	109.5
C13—C14—C15	119.3 (4)	C1—N1—C7	117.7 (3)
C14—C15—C16	120.7 (4)	C1—N1—C10	118.2 (3)
C14—C15—H15	119.6	C7—N1—C10	111.6 (3)
C16—C15—H15	119.6	C8—N2—C9	110.2 (3)
C15—C16—C11	121.0 (4)	C8—N2—H21N	107 (3)
C15—C16—H16	119.5	C9—N2—H21N	112 (3)
C11—C16—H16	119.5	C8—N2—H22N	108 (3)
O3—C17—O4	124.6 (4)	C9—N2—H22N	112 (3)
O3—C17—C11	117.5 (5)	H21N—N2—H22N	107 (4)
O4—C17—C11	117.9 (5)	O1—N3—O2	121.2 (11)
O5—C18—H18C	109.5	O1—N3—C4	118.2 (10)
O5—C18—H18B	109.5	O2—N3—C4	120.6 (9)
H18C—C18—H18B	109.5	O2'—N3'—O1'	122.0 (11)
O5—C18—H18A	109.5	O2'—N3'—C4	119.0 (9)
H18C—C18—H18A	109.5	O1'—N3'—C4	119.0 (10)
H18B—C18—H18A	109.5	C19—N4—C28	121.8 (3)
N4—C19—C20	121.8 (4)	C19—N4—C25	121.4 (4)
N4—C19—C24	122.2 (4)	C28—N4—C25	108.6 (3)
C20—C19—C24	116.0 (4)	C26—N5—C27	112.8 (3)
C21—C20—C19	121.5 (4)	C26—N5—H51N	108 (3)
C21—C20—H20	119.3	C27—N5—H51N	114 (3)
C19—C20—H20	119.3	C26—N5—H52N	107 (3)
C20—C21—C22	121.0 (4)	C27—N5—H52N	112 (3)
C20—C21—H21	119.5	H51N—N5—H52N	103 (4)
C22—C21—H21	119.5	O6—N6—O7	122.1 (4)
C21—C22—C23	120.0 (4)	O6—N6—C22	118.5 (4)
C21—C22—N6	120.4 (4)	O7—N6—C22	119.3 (4)
C23—C22—N6	119.6 (4)	C14—O5—C18	118.7 (4)
C24—C23—C22	119.6 (4)	C32—O10—C36	116.7 (4)
C24—C23—H23	120.2	H11O—O11—H12O	108 (5)
			
N1—C1—C2—C3	−176.3 (4)	O10—C32—C33—C34	−177.5 (4)
C6—C1—C2—C3	1.0 (6)	C31—C32—C33—C34	1.3 (6)
C1—C2—C3—C4	−0.3 (8)	C32—C33—C34—C29	−1.7 (6)
C2—C3—C4—C5	−0.7 (8)	C30—C29—C34—C33	0.7 (6)
C2—C3—C4—N3'	164.9 (6)	C35—C29—C34—C33	179.9 (4)
C2—C3—C4—N3	−165.5 (6)	C30—C29—C35—O9	−4.2 (5)
C3—C4—C5—C6	0.9 (8)	C34—C29—C35—O9	176.6 (3)
N3'—C4—C5—C6	−165.0 (6)	C30—C29—C35—O8	176.7 (3)
N3—C4—C5—C6	165.4 (6)	C34—C29—C35—O8	−2.5 (5)
C4—C5—C6—C1	−0.2 (7)	C6—C1—N1—C7	27.3 (5)
N1—C1—C6—C5	176.6 (4)	C2—C1—N1—C7	−155.5 (4)
C2—C1—C6—C5	−0.7 (6)	C6—C1—N1—C10	166.2 (4)
N1—C7—C8—N2	−54.0 (5)	C2—C1—N1—C10	−16.6 (6)
N2—C9—C10—N1	56.1 (5)	C8—C7—N1—C1	−166.4 (3)
C16—C11—C12—C13	0.2 (6)	C8—C7—N1—C10	52.2 (4)
C17—C11—C12—C13	178.1 (4)	C9—C10—N1—C1	165.3 (3)
C11—C12—C13—C14	−1.6 (7)	C9—C10—N1—C7	−53.4 (5)
C12—C13—C14—O5	−178.3 (4)	C7—C8—N2—C9	55.9 (5)
C12—C13—C14—C15	2.3 (7)	C10—C9—N2—C8	−56.8 (4)
O5—C14—C15—C16	179.0 (4)	C5—C4—N3—O1	5.9 (11)
C13—C14—C15—C16	−1.6 (7)	C3—C4—N3—O1	170.9 (8)
C14—C15—C16—C11	0.1 (7)	C5—C4—N3—O2	−176.8 (7)
C12—C11—C16—C15	0.6 (6)	C3—C4—N3—O2	−11.8 (11)
C17—C11—C16—C15	−177.3 (4)	C5—C4—N3'—O2'	166.2 (8)
C12—C11—C17—O3	−177.7 (4)	C3—C4—N3'—O2'	0.2 (12)
C16—C11—C17—O3	0.1 (6)	C5—C4—N3'—O1'	−15.5 (12)
C12—C11—C17—O4	2.6 (6)	C3—C4—N3'—O1'	178.5 (9)
C16—C11—C17—O4	−179.5 (4)	C20—C19—N4—C28	−18.5 (6)
N4—C19—C20—C21	178.6 (4)	C24—C19—N4—C28	163.8 (4)
C24—C19—C20—C21	−3.6 (6)	C20—C19—N4—C25	−163.3 (4)
C19—C20—C21—C22	1.6 (6)	C24—C19—N4—C25	19.0 (6)
C20—C21—C22—C23	1.1 (6)	C27—C28—N4—C19	−86.8 (5)
C20—C21—C22—N6	−175.8 (4)	C27—C28—N4—C25	61.9 (4)
C21—C22—C23—C24	−1.4 (6)	C26—C25—N4—C19	86.9 (4)
N6—C22—C23—C24	175.5 (4)	C26—C25—N4—C28	−61.9 (4)
C22—C23—C24—C19	−0.8 (6)	C25—C26—N5—C27	−54.1 (5)
N4—C19—C24—C23	−179.0 (4)	C28—C27—N5—C26	54.7 (4)
C20—C19—C24—C23	3.2 (6)	C21—C22—N6—O6	4.5 (6)
N4—C25—C26—N5	57.6 (5)	C23—C22—N6—O6	−172.4 (4)
N5—C27—C28—N4	−58.0 (4)	C21—C22—N6—O7	−175.1 (4)
C34—C29—C30—C31	0.8 (6)	C23—C22—N6—O7	8.0 (6)
C35—C29—C30—C31	−178.5 (4)	C13—C14—O5—C18	0.5 (7)
C29—C30—C31—C32	−1.2 (6)	C15—C14—O5—C18	179.9 (4)
C30—C31—C32—O10	179.1 (4)	C31—C32—O10—C36	176.8 (4)
C30—C31—C32—C33	0.2 (6)	C33—C32—O10—C36	−4.4 (6)

### 4-(4-Nitrophenyl)piperazin-1-ium 4-methoxybenzoate hemihydrate (V) Hydrogen-bond geometry (Å, º)

**Table:**

*D*—H···*A*	*D*—H	H···*A*	*D*···*A*	*D*—H···*A*
C7—H7*B*···O7^i^	0.97	2.54	3.451 (5)	157
C9—H9*B*···O4^ii^	0.97	2.31	3.270 (5)	169
C20—H20···O9	0.93	2.53	3.461 (5)	174
C25—H25*A*···O2a^iii^	0.97	2.5	3.206 (10)	130
C25—H25*A*···O2′b^iii^	0.97	2.49	3.212 (11)	131
C27—H27*A*···O7^i^	0.97	2.58	3.548 (5)	175
C28—H28*B*···O9	0.97	2.55	3.489 (5)	164
C36—H36*C*···O1′b^ii^	0.96	2.49	3.395 (14)	158
N2—H21*N*···O8^iv^	0.88 (2)	1.83 (2)	2.697 (4)	166 (4)
N2—H21*N*···O9^iv^	0.88 (2)	2.57 (3)	3.196 (4)	129 (3)
N2—H22*N*···O11^v^	0.88 (2)	1.89 (2)	2.758 (5)	169 (4)
N5—H51*N*···O9^vi^	0.87 (2)	1.93 (2)	2.778 (5)	164 (4)
N5—H52*N*···O3^vii^	0.91 (2)	1.82 (2)	2.724 (5)	171 (4)
O11—H11*O*···O4	0.84 (2)	1.83 (2)	2.663 (4)	176 (4)
O11—H12*O*···O8^v^	0.84 (2)	1.92 (2)	2.754 (4)	173 (4)

Symmetry codes: (i) −*x*+1, −*y*+1, −*z*; (ii) −*x*+1, *y*+1/2, −*z*+1/2; (iii) *x*, −*y*+1/2, *z*−1/2; (iv) −*x*, −*y*+1, −*z*; (v) −*x*+1, *y*−1/2, −*z*+1/2; (vi) *x*, *y*−1, *z*; (vii) *x*−1, −*y*+1/2, *z*−1/2.

### 4-(4-Nitrophenyl)piperazin-1-ium 4-ethoxybenzoate (VI) Crystal data

**Table:**

C_10_H_14_N_3_O_2_^+^·C_9_H_9_O_3_^−^	*Z* = 4
*M**_r_* = 373.4	*F*(000) = 792
Triclinic, *P*1	*D*_x_ = 1.324 Mg m^−^^3^
Hall symbol: -P 1	Mo *K*α radiation, λ = 0.71073 Å
*a* = 7.874 (1) Å	Cell parameters from 4134 reflections
*b* = 9.263 (1) Å	θ = 2.4–28.0°
*c* = 27.996 (3) Å	µ = 0.10 mm^−^^1^
α = 81.030 (6)°	*T* = 293 K
β = 85.675 (6)°	Plate, yellow
γ = 68.229 (5)°	0.44 × 0.32 × 0.08 mm
*V* = 1872.8 (4) Å^3^	

### 4-(4-Nitrophenyl)piperazin-1-ium 4-ethoxybenzoate (VI) Data collection

**Table:**

Oxford Diffraction Xcalibur diffractometer	3803 reflections with *I* > 2σ(*I*)
ω scans	*R*_int_ = 0.027
Absorption correction: multi-scan (CrysAlis RED; Oxford Diffraction, 2009)	θ_max_ = 25.4°, θ_min_ = 2.4°
*T*_min_ = 0.963, *T*_max_ = 0.992	*h* = −9→5
13344 measured reflections	*k* = −11→10
6868 independent reflections	*l* = −33→33

### 4-(4-Nitrophenyl)piperazin-1-ium 4-ethoxybenzoate (VI) Refinement

**Table:**

Refinement on *F*^2^	0 constraints
Least-squares matrix: full	Hydrogen site location: mixed
*R*[*F*^2^ > 2σ(*F*^2^)] = 0.061	H atoms treated by a mixture of independent and constrained refinement
*wR*(*F*^2^) = 0.137	*w* = 1/[σ^2^(*F*_o_^2^) + (0.0432*P*)^2^ + 0.7484*P*] where *P* = (*F*_o_^2^ + 2*F*_c_^2^)/3
*S* = 1.05	(Δ/σ)_max_ < 0.001
6858 reflections	Δρ_max_ = 0.23 e Å^−^^3^
501 parameters	Δρ_min_ = −0.22 e Å^−^^3^
16 restraints	

### 4-(4-Nitrophenyl)piperazin-1-ium 4-ethoxybenzoate (VI) Special details

**Table:**

Geometry. All esds (except the esd in the dihedral angle between two l.s. planes) are estimated using the full covariance matrix. The cell esds are taken into account individually in the estimation of esds in distances, angles and torsion angles; correlations between esds in cell parameters are only used when they are defined by crystal symmetry. An approximate (isotropic) treatment of cell esds is used for estimating esds involving l.s. planes.

### 4-(4-Nitrophenyl)piperazin-1-ium 4-ethoxybenzoate (VI) Fractional atomic coordinates and isotropic or equivalent isotropic displacement parameters (Å^2^)

**Table:**

	*x*	*y*	*z*	*U*_iso_*/*U*_eq_	
O1	−0.3466 (3)	0.6545 (4)	0.40786 (9)	0.1126 (10)	
O2	−0.1630 (3)	0.5811 (3)	0.46683 (9)	0.0901 (8)	
N3	−0.1931 (4)	0.6107 (3)	0.42384 (10)	0.0667 (7)	
N1	0.3914 (3)	0.5404 (2)	0.29014 (7)	0.0412 (5)	
N2	0.6838 (3)	0.4199 (3)	0.22275 (9)	0.0486 (6)	
C1	0.2485 (3)	0.5547 (3)	0.32330 (9)	0.0364 (6)	
C2	0.0659 (4)	0.6276 (3)	0.30870 (10)	0.0452 (7)	
H2	0.041054	0.663561	0.276117	0.054*	
C3	−0.0759 (4)	0.6467 (3)	0.34138 (10)	0.0487 (7)	
H3	−0.195824	0.69631	0.330993	0.058*	
C4	−0.0417 (4)	0.5927 (3)	0.38966 (10)	0.0470 (7)	
C5	0.1352 (4)	0.5197 (3)	0.40558 (10)	0.0460 (7)	
H5	0.157333	0.483265	0.438246	0.055*	
C6	0.2787 (4)	0.5009 (3)	0.37309 (10)	0.0430 (7)	
H6	0.397837	0.451799	0.384052	0.052*	
C7	0.5772 (3)	0.4477 (3)	0.30663 (10)	0.0473 (7)	
H7A	0.588757	0.338899	0.315234	0.057*	
H7B	0.597413	0.484321	0.33554	0.057*	
C8	0.7216 (4)	0.4565 (4)	0.26954 (10)	0.0539 (8)	
H8A	0.725864	0.561056	0.265102	0.065*	
H8B	0.839991	0.382613	0.280859	0.065*	
C9	0.5080 (4)	0.5395 (3)	0.20545 (10)	0.0528 (8)	
H9A	0.482935	0.520499	0.174084	0.063*	
H9B	0.516375	0.642512	0.201592	0.063*	
C10	0.3546 (4)	0.5366 (4)	0.23985 (9)	0.0527 (8)	
H10A	0.245506	0.626087	0.229683	0.063*	
H10B	0.329888	0.442345	0.238274	0.063*	
O3	−0.0728 (3)	0.4331 (2)	0.15512 (7)	0.0578 (5)	
O4	0.1149 (2)	0.2978 (2)	0.21489 (7)	0.0532 (5)	
O5	0.6778 (3)	0.3001 (3)	0.03965 (7)	0.0734 (7)	
C11	0.2434 (4)	0.3357 (3)	0.13647 (9)	0.0400 (6)	
C12	0.4193 (4)	0.2483 (3)	0.15096 (10)	0.0518 (8)	
H12	0.438717	0.198421	0.182667	0.062*	
C13	0.5679 (4)	0.2321 (4)	0.11995 (10)	0.0572 (8)	
H13	0.685415	0.171644	0.130663	0.069*	
C14	0.5409 (4)	0.3059 (3)	0.07314 (10)	0.0524 (8)	
C15	0.3656 (4)	0.3930 (4)	0.05764 (10)	0.0609 (9)	
H15	0.346406	0.442258	0.02587	0.073*	
C16	0.2192 (4)	0.4072 (3)	0.08898 (10)	0.0520 (8)	
H16	0.101521	0.466057	0.078056	0.062*	
C17	0.0851 (4)	0.3564 (3)	0.17148 (10)	0.0424 (7)	
C18	0.8615 (4)	0.2090 (4)	0.05351 (12)	0.0820 (11)	
H18A	0.874909	0.100553	0.064059	0.098*	
H18B	0.894362	0.249403	0.079923	0.098*	
C19	0.9824 (5)	0.2199 (5)	0.01002 (14)	0.1126 (16)	
H19A	1.106148	0.152009	0.017216	0.169*	
H19B	0.976652	0.32615	0.001769	0.169*	
H19C	0.941525	0.188565	−0.016723	0.169*	
O6	0.1516 (4)	0.7336 (4)	0.60499 (10)	0.1157 (11)	
O7	0.1799 (4)	0.9508 (4)	0.61439 (10)	0.1162 (11)	
N6	0.1845 (4)	0.8525 (5)	0.58929 (11)	0.0830 (10)	
N4	0.2839 (3)	0.9838 (3)	0.38867 (8)	0.0490 (6)	
N5	0.2937 (3)	1.0699 (3)	0.28629 (8)	0.0462 (6)	
C20	0.2721 (4)	0.9472 (3)	0.43812 (10)	0.0459 (7)	
C21	0.2534 (4)	0.8084 (4)	0.45928 (11)	0.0653 (9)	
H21	0.259467	0.733784	0.439839	0.078*	
C22	0.2260 (5)	0.7782 (4)	0.50814 (12)	0.0747 (10)	
H22	0.211482	0.684754	0.521174	0.09*	
C23	0.2199 (4)	0.8824 (5)	0.53759 (11)	0.0636 (9)	
C24	0.2430 (5)	1.0184 (5)	0.51872 (13)	0.0783 (11)	
H24	0.240692	1.089852	0.538938	0.094*	
C25	0.2700 (5)	1.0504 (4)	0.46951 (12)	0.0711 (10)	
H25	0.287119	1.143254	0.457048	0.085*	
C26	0.3867 (4)	1.0795 (3)	0.36697 (10)	0.0563 (8)	
H26A	0.51213	1.012589	0.360971	0.068*	
H26B	0.3881	1.149872	0.389107	0.068*	
C27	0.3030 (4)	1.1732 (3)	0.32039 (10)	0.0539 (8)	
H27A	0.180752	1.245608	0.326783	0.065*	
H27B	0.375328	1.234293	0.305927	0.065*	
C28	0.1907 (4)	0.9701 (3)	0.30890 (10)	0.0498 (7)	
H28A	0.193308	0.897196	0.28723	0.06*	
H28B	0.064102	1.035801	0.314055	0.06*	
C29	0.2714 (4)	0.8803 (3)	0.35626 (10)	0.0529 (8)	
H29A	0.196135	0.822751	0.371369	0.064*	
H29B	0.392544	0.804857	0.35057	0.064*	
O8	−0.3777 (3)	0.8954 (2)	0.25237 (8)	0.0629 (6)	
O9	−0.3341 (3)	1.1185 (2)	0.25354 (7)	0.0569 (5)	
O10	0.2958 (3)	0.8011 (3)	0.10557 (8)	0.0771 (7)	
C30	−0.1331 (3)	0.9365 (3)	0.20503 (9)	0.0408 (6)	
C31	−0.1058 (4)	0.8152 (3)	0.17834 (10)	0.0508 (7)	
H31	−0.185001	0.760423	0.183038	0.061*	
C32	0.0357 (4)	0.7741 (3)	0.14506 (11)	0.0555 (8)	
H32	0.049421	0.694181	0.127006	0.067*	
C33	0.1575 (4)	0.8517 (3)	0.13846 (10)	0.0511 (7)	
C34	0.1341 (4)	0.9716 (3)	0.16508 (10)	0.0512 (7)	
H34	0.215362	1.024354	0.161223	0.061*	
C35	−0.0111 (4)	1.0121 (3)	0.19741 (10)	0.0463 (7)	
H35	−0.027019	1.09415	0.214785	0.056*	
C36	−0.2919 (4)	0.9871 (4)	0.23973 (10)	0.0478 (7)	
C37	0.4280 (5)	0.8751 (5)	0.09807 (14)	0.0885 (12)	
H37A	0.483742	0.868761	0.128435	0.106*	
H37B	0.369923	0.984835	0.085011	0.106*	
C38	0.5698 (6)	0.7907 (7)	0.06316 (18)	0.153 (2)	
H38A	0.667443	0.830217	0.059976	0.229*	
H38B	0.515701	0.807254	0.032204	0.229*	
H38C	0.617246	0.680406	0.074893	0.229*	
H31N	0.778 (5)	0.425 (6)	0.2007 (14)	0.183*	
H32N	0.677 (7)	0.324 (3)	0.2290 (18)	0.183*	
H61N	0.407 (4)	1.009 (5)	0.2766 (18)	0.183*	
H62N	0.236 (6)	1.131 (5)	0.2599 (12)	0.183*	

### 4-(4-Nitrophenyl)piperazin-1-ium 4-ethoxybenzoate (VI) Atomic displacement parameters (Å^2^)

**Table:**

	*U*^11^	*U*^22^	*U*^33^	*U*^12^	*U*^13^	*U*^23^
O1	0.0514 (15)	0.173 (3)	0.0801 (19)	−0.0138 (17)	0.0103 (14)	0.0040 (18)
O2	0.0855 (17)	0.129 (2)	0.0445 (15)	−0.0320 (16)	0.0107 (13)	−0.0023 (14)
N3	0.0550 (18)	0.076 (2)	0.0573 (19)	−0.0143 (15)	0.0078 (15)	−0.0036 (15)
N1	0.0399 (13)	0.0448 (14)	0.0380 (13)	−0.0148 (11)	−0.0030 (10)	−0.0033 (11)
N2	0.0450 (14)	0.0536 (16)	0.0466 (15)	−0.0175 (13)	0.0038 (11)	−0.0089 (13)
C1	0.0440 (16)	0.0302 (15)	0.0369 (16)	−0.0153 (13)	−0.0019 (13)	−0.0053 (12)
C2	0.0486 (17)	0.0463 (17)	0.0378 (16)	−0.0152 (14)	−0.0064 (14)	−0.0001 (13)
C3	0.0392 (16)	0.0525 (19)	0.0513 (19)	−0.0132 (14)	−0.0007 (14)	−0.0071 (15)
C4	0.0488 (18)	0.0463 (18)	0.0441 (18)	−0.0167 (15)	0.0066 (14)	−0.0065 (14)
C5	0.0584 (19)	0.0406 (17)	0.0386 (16)	−0.0186 (15)	−0.0016 (14)	−0.0020 (13)
C6	0.0433 (16)	0.0381 (16)	0.0457 (17)	−0.0127 (13)	−0.0066 (14)	−0.0027 (13)
C7	0.0407 (16)	0.0543 (18)	0.0470 (18)	−0.0162 (14)	−0.0043 (13)	−0.0088 (14)
C8	0.0440 (17)	0.066 (2)	0.057 (2)	−0.0243 (15)	0.0013 (15)	−0.0150 (16)
C9	0.0544 (18)	0.0547 (19)	0.0451 (18)	−0.0186 (16)	0.0029 (14)	0.0005 (14)
C10	0.0452 (17)	0.068 (2)	0.0392 (17)	−0.0168 (15)	−0.0010 (13)	−0.0005 (15)
O3	0.0458 (12)	0.0732 (15)	0.0477 (12)	−0.0181 (11)	0.0023 (10)	0.0009 (11)
O4	0.0585 (12)	0.0634 (13)	0.0375 (12)	−0.0259 (11)	0.0003 (9)	0.0022 (10)
O5	0.0505 (13)	0.0959 (17)	0.0504 (13)	−0.0107 (12)	0.0086 (11)	0.0129 (12)
C11	0.0480 (17)	0.0392 (16)	0.0352 (15)	−0.0199 (14)	0.0001 (13)	−0.0026 (13)
C12	0.0524 (18)	0.064 (2)	0.0362 (16)	−0.0234 (16)	−0.0022 (14)	0.0074 (14)
C13	0.0453 (17)	0.072 (2)	0.0455 (19)	−0.0155 (16)	−0.0029 (15)	0.0053 (16)
C14	0.0489 (18)	0.060 (2)	0.0424 (18)	−0.0173 (16)	0.0060 (15)	−0.0001 (15)
C15	0.056 (2)	0.075 (2)	0.0327 (17)	−0.0089 (17)	0.0009 (15)	0.0090 (15)
C16	0.0458 (17)	0.0560 (19)	0.0431 (18)	−0.0091 (15)	−0.0006 (14)	0.0016 (15)
C17	0.0498 (18)	0.0416 (17)	0.0418 (18)	−0.0237 (15)	0.0000 (14)	−0.0054 (14)
C18	0.050 (2)	0.106 (3)	0.069 (2)	−0.014 (2)	0.0095 (17)	0.007 (2)
C19	0.058 (2)	0.154 (4)	0.088 (3)	−0.011 (2)	0.022 (2)	0.011 (3)
O6	0.125 (3)	0.136 (3)	0.0580 (18)	−0.028 (2)	0.0049 (16)	0.0186 (18)
O7	0.099 (2)	0.208 (3)	0.0619 (18)	−0.070 (2)	0.0107 (15)	−0.049 (2)
N6	0.0584 (19)	0.129 (3)	0.049 (2)	−0.022 (2)	0.0005 (15)	−0.010 (2)
N4	0.0744 (17)	0.0449 (14)	0.0381 (14)	−0.0343 (13)	−0.0005 (12)	−0.0046 (11)
N5	0.0553 (15)	0.0425 (15)	0.0435 (14)	−0.0231 (12)	0.0049 (12)	−0.0033 (12)
C20	0.0459 (16)	0.0463 (18)	0.0443 (18)	−0.0147 (14)	0.0001 (13)	−0.0083 (14)
C21	0.099 (3)	0.054 (2)	0.0440 (19)	−0.0301 (19)	0.0055 (17)	−0.0063 (16)
C22	0.101 (3)	0.070 (2)	0.047 (2)	−0.031 (2)	0.0048 (19)	0.0032 (18)
C23	0.0510 (19)	0.091 (3)	0.0396 (19)	−0.0177 (19)	−0.0027 (15)	−0.0030 (19)
C24	0.087 (3)	0.110 (3)	0.051 (2)	−0.041 (2)	0.0054 (19)	−0.038 (2)
C25	0.099 (3)	0.074 (2)	0.059 (2)	−0.049 (2)	0.0046 (19)	−0.0196 (19)
C26	0.071 (2)	0.058 (2)	0.0524 (19)	−0.0381 (17)	0.0000 (16)	−0.0085 (16)
C27	0.072 (2)	0.0427 (17)	0.0533 (19)	−0.0303 (16)	0.0104 (16)	−0.0060 (15)
C28	0.0607 (19)	0.0526 (18)	0.0439 (18)	−0.0310 (16)	0.0008 (14)	−0.0043 (14)
C29	0.077 (2)	0.0475 (18)	0.0436 (18)	−0.0344 (16)	−0.0019 (15)	−0.0032 (14)
O8	0.0597 (13)	0.0531 (13)	0.0781 (15)	−0.0273 (11)	0.0142 (11)	−0.0062 (11)
O9	0.0627 (13)	0.0524 (13)	0.0573 (13)	−0.0226 (11)	0.0088 (10)	−0.0129 (11)
O10	0.0744 (15)	0.0862 (17)	0.0732 (16)	−0.0304 (14)	0.0256 (13)	−0.0281 (13)
C30	0.0447 (16)	0.0366 (16)	0.0382 (16)	−0.0133 (13)	−0.0061 (13)	0.0021 (13)
C31	0.0524 (18)	0.0469 (18)	0.0559 (19)	−0.0229 (15)	−0.0043 (15)	−0.0020 (15)
C32	0.064 (2)	0.0469 (18)	0.056 (2)	−0.0192 (16)	−0.0013 (16)	−0.0139 (15)
C33	0.0507 (18)	0.0488 (18)	0.0463 (18)	−0.0113 (15)	0.0020 (14)	−0.0032 (15)
C34	0.0482 (17)	0.0498 (19)	0.058 (2)	−0.0232 (15)	0.0023 (15)	−0.0036 (16)
C35	0.0490 (17)	0.0437 (17)	0.0490 (18)	−0.0186 (14)	−0.0048 (14)	−0.0077 (14)
C36	0.0480 (18)	0.0481 (19)	0.0442 (18)	−0.0162 (15)	−0.0073 (14)	0.0022 (15)
C37	0.075 (3)	0.110 (3)	0.082 (3)	−0.040 (2)	0.025 (2)	−0.014 (2)
C38	0.122 (4)	0.205 (6)	0.147 (5)	−0.072 (4)	0.083 (4)	−0.079 (4)

### 4-(4-Nitrophenyl)piperazin-1-ium 4-ethoxybenzoate (VI) Geometric parameters (Å, º)

**Table:**

O1—N3	1.220 (3)	O6—N6	1.231 (4)
O2—N3	1.213 (3)	O7—N6	1.223 (4)
N3—C4	1.447 (3)	N6—C23	1.456 (4)
N1—C1	1.384 (3)	N4—C20	1.378 (3)
N1—C7	1.462 (3)	N4—C26	1.449 (3)
N1—C10	1.467 (3)	N4—C29	1.452 (3)
N2—C9	1.475 (3)	N5—C27	1.476 (4)
N2—C8	1.481 (4)	N5—C28	1.486 (3)
N2—H31N	0.937 (19)	N5—H61N	0.910 (19)
N2—H32N	0.895 (19)	N5—H62N	0.896 (19)
C1—C2	1.405 (3)	C20—C21	1.384 (4)
C1—C6	1.412 (3)	C20—C25	1.391 (4)
C2—C3	1.366 (4)	C21—C22	1.370 (4)
C2—H2	0.93	C21—H21	0.93
C3—C4	1.376 (4)	C22—C23	1.349 (4)
C3—H3	0.93	C22—H22	0.93
C4—C5	1.377 (4)	C23—C24	1.359 (5)
C5—C6	1.372 (3)	C24—C25	1.381 (4)
C5—H5	0.93	C24—H24	0.93
C6—H6	0.93	C25—H25	0.93
C7—C8	1.498 (3)	C26—C27	1.497 (4)
C7—H7A	0.97	C26—H26A	0.97
C7—H7B	0.97	C26—H26B	0.97
C8—H8A	0.97	C27—H27A	0.97
C8—H8B	0.97	C27—H27B	0.97
C9—C10	1.493 (3)	C28—C29	1.498 (4)
C9—H9A	0.97	C28—H28A	0.97
C9—H9B	0.97	C28—H28B	0.97
C10—H10A	0.97	C29—H29A	0.97
C10—H10B	0.97	C29—H29B	0.97
O3—C17	1.261 (3)	O8—C36	1.264 (3)
O4—C17	1.253 (3)	O9—C36	1.252 (3)
O5—C14	1.365 (3)	O10—C33	1.363 (3)
O5—C18	1.426 (3)	O10—C37	1.431 (4)
C11—C12	1.375 (4)	C30—C35	1.372 (3)
C11—C16	1.383 (3)	C30—C31	1.387 (4)
C11—C17	1.499 (4)	C30—C36	1.500 (4)
C12—C13	1.379 (4)	C31—C32	1.376 (4)
C12—H12	0.93	C31—H31	0.93
C13—C14	1.373 (4)	C32—C33	1.383 (4)
C13—H13	0.93	C32—H32	0.93
C14—C15	1.378 (4)	C33—C34	1.381 (4)
C15—C16	1.374 (4)	C34—C35	1.378 (4)
C15—H15	0.93	C34—H34	0.93
C16—H16	0.93	C35—H35	0.93
C18—C19	1.502 (4)	C37—C38	1.496 (5)
C18—H18A	0.97	C37—H37A	0.97
C18—H18B	0.97	C37—H37B	0.97
C19—H19A	0.96	C38—H38A	0.96
C19—H19B	0.96	C38—H38B	0.96
C19—H19C	0.96	C38—H38C	0.96
			
O2—N3—O1	122.7 (3)	O7—N6—O6	123.5 (4)
O2—N3—C4	119.3 (3)	O7—N6—C23	118.2 (4)
O1—N3—C4	118.0 (3)	O6—N6—C23	118.2 (4)
C1—N1—C7	118.1 (2)	C20—N4—C26	121.3 (2)
C1—N1—C10	117.0 (2)	C20—N4—C29	121.3 (2)
C7—N1—C10	116.3 (2)	C26—N4—C29	111.9 (2)
C9—N2—C8	107.9 (2)	C27—N5—C28	110.0 (2)
C9—N2—H31N	110 (3)	C27—N5—H61N	112 (3)
C8—N2—H31N	108 (3)	C28—N5—H61N	110 (3)
C9—N2—H32N	111 (3)	C27—N5—H62N	108 (3)
C8—N2—H32N	106 (3)	C28—N5—H62N	110 (3)
H31N—N2—H32N	113 (4)	H61N—N5—H62N	107 (4)
N1—C1—C2	120.9 (2)	N4—C20—C21	121.8 (3)
N1—C1—C6	122.0 (2)	N4—C20—C25	122.0 (3)
C2—C1—C6	117.1 (2)	C21—C20—C25	116.2 (3)
C3—C2—C1	121.3 (3)	C22—C21—C20	121.6 (3)
C3—C2—H2	119.3	C22—C21—H21	119.2
C1—C2—H2	119.3	C20—C21—H21	119.2
C2—C3—C4	120.1 (3)	C23—C22—C21	120.9 (3)
C2—C3—H3	120	C23—C22—H22	119.6
C4—C3—H3	120	C21—C22—H22	119.6
C3—C4—C5	120.6 (3)	C22—C23—C24	119.7 (3)
C3—C4—N3	119.6 (3)	C22—C23—N6	120.8 (4)
C5—C4—N3	119.8 (3)	C24—C23—N6	119.5 (4)
C6—C5—C4	119.8 (3)	C23—C24—C25	119.9 (3)
C6—C5—H5	120.1	C23—C24—H24	120
C4—C5—H5	120.1	C25—C24—H24	120
C5—C6—C1	121.2 (3)	C24—C25—C20	121.6 (3)
C5—C6—H6	119.4	C24—C25—H25	119.2
C1—C6—H6	119.4	C20—C25—H25	119.2
N1—C7—C8	113.3 (2)	N4—C26—C27	110.5 (2)
N1—C7—H7A	108.9	N4—C26—H26A	109.5
C8—C7—H7A	108.9	C27—C26—H26A	109.5
N1—C7—H7B	108.9	N4—C26—H26B	109.5
C8—C7—H7B	108.9	C27—C26—H26B	109.5
H7A—C7—H7B	107.7	H26A—C26—H26B	108.1
N2—C8—C7	110.9 (2)	N5—C27—C26	111.0 (2)
N2—C8—H8A	109.5	N5—C27—H27A	109.4
C7—C8—H8A	109.5	C26—C27—H27A	109.4
N2—C8—H8B	109.5	N5—C27—H27B	109.4
C7—C8—H8B	109.5	C26—C27—H27B	109.4
H8A—C8—H8B	108	H27A—C27—H27B	108
N2—C9—C10	111.5 (2)	N5—C28—C29	111.1 (2)
N2—C9—H9A	109.3	N5—C28—H28A	109.4
C10—C9—H9A	109.3	C29—C28—H28A	109.4
N2—C9—H9B	109.3	N5—C28—H28B	109.4
C10—C9—H9B	109.3	C29—C28—H28B	109.4
H9A—C9—H9B	108	H28A—C28—H28B	108
N1—C10—C9	113.7 (2)	N4—C29—C28	111.5 (2)
N1—C10—H10A	108.8	N4—C29—H29A	109.3
C9—C10—H10A	108.8	C28—C29—H29A	109.3
N1—C10—H10B	108.8	N4—C29—H29B	109.3
C9—C10—H10B	108.8	C28—C29—H29B	109.3
H10A—C10—H10B	107.7	H29A—C29—H29B	108
C14—O5—C18	118.3 (2)	C33—O10—C37	118.5 (3)
C12—C11—C16	117.5 (2)	C35—C30—C31	117.1 (3)
C12—C11—C17	120.9 (2)	C35—C30—C36	120.9 (3)
C16—C11—C17	121.6 (3)	C31—C30—C36	122.0 (2)
C11—C12—C13	122.1 (3)	C32—C31—C30	121.5 (3)
C11—C12—H12	119	C32—C31—H31	119.2
C13—C12—H12	119	C30—C31—H31	119.2
C14—C13—C12	119.5 (3)	C31—C32—C33	120.0 (3)
C14—C13—H13	120.3	C31—C32—H32	120
C12—C13—H13	120.3	C33—C32—H32	120
O5—C14—C13	124.4 (3)	O10—C33—C34	124.5 (3)
O5—C14—C15	116.1 (3)	O10—C33—C32	116.1 (3)
C13—C14—C15	119.5 (3)	C34—C33—C32	119.4 (3)
C16—C15—C14	120.2 (3)	C35—C34—C33	119.2 (3)
C16—C15—H15	119.9	C35—C34—H34	120.4
C14—C15—H15	119.9	C33—C34—H34	120.4
C15—C16—C11	121.2 (3)	C30—C35—C34	122.7 (3)
C15—C16—H16	119.4	C30—C35—H35	118.6
C11—C16—H16	119.4	C34—C35—H35	118.6
O4—C17—O3	123.5 (2)	O9—C36—O8	124.0 (3)
O4—C17—C11	119.3 (3)	O9—C36—C30	118.3 (3)
O3—C17—C11	117.3 (2)	O8—C36—C30	117.7 (3)
O5—C18—C19	107.3 (3)	O10—C37—C38	107.4 (3)
O5—C18—H18A	110.3	O10—C37—H37A	110.2
C19—C18—H18A	110.3	C38—C37—H37A	110.2
O5—C18—H18B	110.3	O10—C37—H37B	110.2
C19—C18—H18B	110.3	C38—C37—H37B	110.2
H18A—C18—H18B	108.5	H37A—C37—H37B	108.5
C18—C19—H19A	109.5	C37—C38—H38A	109.5
C18—C19—H19B	109.5	C37—C38—H38B	109.5
H19A—C19—H19B	109.5	H38A—C38—H38B	109.5
C18—C19—H19C	109.5	C37—C38—H38C	109.5
H19A—C19—H19C	109.5	H38A—C38—H38C	109.5
H19B—C19—H19C	109.5	H38B—C38—H38C	109.5
			
C7—N1—C1—C2	−173.1 (2)	C26—N4—C20—C21	−149.7 (3)
C10—N1—C1—C2	−26.5 (3)	C29—N4—C20—C21	2.0 (4)
C7—N1—C1—C6	8.5 (3)	C26—N4—C20—C25	33.3 (4)
C10—N1—C1—C6	155.0 (2)	C29—N4—C20—C25	−175.1 (3)
N1—C1—C2—C3	−177.9 (2)	N4—C20—C21—C22	−174.3 (3)
C6—C1—C2—C3	0.6 (4)	C25—C20—C21—C22	2.8 (5)
C1—C2—C3—C4	−0.7 (4)	C20—C21—C22—C23	−1.3 (5)
C2—C3—C4—C5	0.3 (4)	C21—C22—C23—C24	−0.7 (5)
C2—C3—C4—N3	−178.7 (3)	C21—C22—C23—N6	177.8 (3)
O2—N3—C4—C3	−170.8 (3)	O7—N6—C23—C22	−179.8 (3)
O1—N3—C4—C3	9.6 (4)	O6—N6—C23—C22	−3.4 (5)
O2—N3—C4—C5	10.2 (4)	O7—N6—C23—C24	−1.3 (5)
O1—N3—C4—C5	−169.4 (3)	O6—N6—C23—C24	175.1 (3)
C3—C4—C5—C6	0.2 (4)	C22—C23—C24—C25	0.9 (5)
N3—C4—C5—C6	179.2 (3)	N6—C23—C24—C25	−177.6 (3)
C4—C5—C6—C1	−0.2 (4)	C23—C24—C25—C20	0.8 (5)
N1—C1—C6—C5	178.3 (2)	N4—C20—C25—C24	174.6 (3)
C2—C1—C6—C5	−0.2 (4)	C21—C20—C25—C24	−2.6 (5)
C1—N1—C7—C8	−173.0 (2)	C20—N4—C26—C27	−148.9 (3)
C10—N1—C7—C8	40.3 (3)	C29—N4—C26—C27	57.0 (3)
C9—N2—C8—C7	62.2 (3)	C28—N5—C27—C26	56.4 (3)
N1—C7—C8—N2	−51.8 (3)	N4—C26—C27—N5	−57.5 (3)
C8—N2—C9—C10	−61.5 (3)	C27—N5—C28—C29	−54.8 (3)
C1—N1—C10—C9	173.4 (2)	C20—N4—C29—C28	150.1 (2)
C7—N1—C10—C9	−39.5 (3)	C26—N4—C29—C28	−55.8 (3)
N2—C9—C10—N1	50.3 (3)	N5—C28—C29—N4	54.6 (3)
C16—C11—C12—C13	0.5 (4)	C35—C30—C31—C32	−1.0 (4)
C17—C11—C12—C13	−177.6 (3)	C36—C30—C31—C32	177.1 (3)
C11—C12—C13—C14	0.4 (5)	C30—C31—C32—C33	1.6 (4)
C18—O5—C14—C13	1.3 (5)	C37—O10—C33—C34	0.7 (4)
C18—O5—C14—C15	−178.7 (3)	C37—O10—C33—C32	−178.7 (3)
C12—C13—C14—O5	178.9 (3)	C31—C32—C33—O10	178.6 (3)
C12—C13—C14—C15	−1.0 (5)	C31—C32—C33—C34	−0.8 (4)
O5—C14—C15—C16	−179.2 (3)	O10—C33—C34—C35	−179.8 (3)
C13—C14—C15—C16	0.7 (5)	C32—C33—C34—C35	−0.5 (4)
C14—C15—C16—C11	0.2 (5)	C31—C30—C35—C34	−0.3 (4)
C12—C11—C16—C15	−0.8 (4)	C36—C30—C35—C34	−178.5 (3)
C17—C11—C16—C15	177.3 (3)	C33—C34—C35—C30	1.1 (4)
C12—C11—C17—O4	2.7 (4)	C35—C30—C36—O9	15.2 (4)
C16—C11—C17—O4	−175.3 (2)	C31—C30—C36—O9	−162.9 (3)
C12—C11—C17—O3	−177.3 (3)	C35—C30—C36—O8	−166.4 (2)
C16—C11—C17—O3	4.6 (4)	C31—C30—C36—O8	15.5 (4)
C14—O5—C18—C19	178.7 (3)	C33—O10—C37—C38	176.2 (3)

### 4-(4-Nitrophenyl)piperazin-1-ium 4-ethoxybenzoate (VI) Hydrogen-bond geometry (Å, º)

*Cg*2 and *Cg*6 are the centroids of the C1–C6 and C30–C35 rings, respectively.

**Table:**

*D*—H···*A*	*D*—H	H···*A*	*D*···*A*	*D*—H···*A*
N2—H31*N*···O3^i^	0.94 (2)	1.68 (2)	2.613 (3)	172 (5)
N2—H31*N*···O4^i^	0.94 (2)	2.51 (4)	3.157 (3)	127 (4)
N2—H32*N*···O9^ii^	0.90 (2)	1.96 (2)	2.843 (3)	171 (5)
N5—H61*N*···O8^i^	0.91 (2)	1.78 (2)	2.686 (3)	175 (5)
N5—H61*N*···O9^i^	0.91 (2)	2.59 (4)	3.174 (3)	122 (4)
N5—H62*N*···O4^iii^	0.90 (2)	1.83 (2)	2.708 (3)	165 (5)
C22—H22···O2^iv^	0.93	2.6	3.502 (5)	165
C27—H27*B*···O9^i^	0.97	2.59	3.215 (3)	123
C28—H28*B*···O7^v^	0.97	2.65	3.410 (4)	135
C29—H29*B*···O1^i^	0.97	2.53	3.249 (4)	131
C35—H35···O4^iii^	0.93	2.52	3.263 (3)	137
C10—H10*A*···*Cg*6	0.97	2.82	3.746 (3)	159
C29—H29*A*···*Cg*2	0.97	2.76	3.556 (3)	139

Symmetry codes: (i) *x*+1, *y*, *z*; (ii) *x*+1, *y*−1, *z*; (iii) *x*, *y*+1, *z*; (iv) −*x*, −*y*+1, −*z*+1; (v) −*x*, −*y*+2, −*z*+1.
